# Chemotherapy-induced COX-2 upregulation by cancer cells defines their inflammatory properties and limits the efficacy of chemoimmunotherapy combinations

**DOI:** 10.1038/s41467-022-29606-9

**Published:** 2022-04-19

**Authors:** Charlotte R. Bell, Victoria S. Pelly, Agrin Moeini, Shih-Chieh Chiang, Eimear Flanagan, Christian P. Bromley, Christopher Clark, Charles H. Earnshaw, Maria A. Koufaki, Eduardo Bonavita, Santiago Zelenay

**Affiliations:** 1 Cancer Inflammation and Immunity Group, Cancer Research UK Manchester Institute, The University of Manchester, Alderley Park, Manchester, UK; 2 Molecular Biology Core Facility, Cancer Research UK Manchester Institute, The University of Manchester, Alderley Park, Manchester, UK; 3 The Lydia Becker Institute of Immunology and Inflammation, The University of Manchester, Manchester, UK

**Keywords:** Tumour immunology, Cancer immunotherapy, Tumour immunology

## Abstract

Cytotoxic therapies, besides directly inducing cancer cell death, can stimulate immune-dependent tumor growth control or paradoxically accelerate tumor progression. The underlying mechanisms dictating these opposing outcomes are poorly defined. Here, we show that cytotoxic therapy acutely upregulates cyclooxygenase (COX)-2 expression and prostaglandin E_2_ (PGE_2_) production in cancer cells with pre-existing COX-2 activity. Screening a compound library of 1280 approved drugs, we find that all classes of chemotherapy drugs enhance COX-2 transcription whilst arresting cancer cell proliferation. Genetic manipulation of COX-2 expression or its gene promoter region uncover how augmented COX-2/PGE_2_ activity post-treatment profoundly alters the inflammatory properties of chemotherapy-treated cancer cells in vivo. Pharmacological COX-2 inhibition boosts the efficacy of the combination of chemotherapy and PD-1 blockade. Crucially, in a poorly immunogenic breast cancer model, only the triple therapy unleashes tumor growth control and significantly reduces relapse and spontaneous metastatic spread in an adjuvant setting. Our findings suggest COX-2/PGE_2_ upregulation by dying cancer cells acts as a major barrier to cytotoxic therapy-driven tumor immunity and uncover a strategy to improve the outcomes of immunotherapy and chemotherapy combinations.

## Abstract (web summary)

COX-2-mediated prostaglandin E2 (PGE2) release from dying cancer cells contributes to cytotoxic therapy resistance. Here the authors show that cytotoxic drugs induce PGE2 release only in cancer cells with basal COX-2 activity and that pharmacological COX-2 inhibition can boost the efficacy of the combination of chemotherapy and PD-1 blockade.

## Introduction

Immune checkpoint blockade (ICB) therapies targeting the cytotoxic T lymphocyte-associated antigen-4 and programmed cell death (PD)−1 pathways have transformed the landscape of cancer treatment^1^. Despite these advances, cytotoxic therapies, such as chemotherapy (CTX) or radiotherapy, remain the standard of care for most unresectable or advanced malignancies, including in adjuvant and neoadjuvant settings.

Tumor shrinkage following CTX and radiotherapy has been largely attributed to the damaging effects of these cytotoxic agents on rapidly proliferating cancer cells. In addition to their direct killing of cancer cells, numerous studies have also highlighted a major role for the immune system in mediating the efficacy of these therapies^2,3^. Hallmark cellular and molecular mediators of anti-cancer immune responses are indispensable for, and correlate with, the efficacy of cytotoxic therapy in animal models and human cancers, respectively^3^. These observations are consistent with the view that release of damage-associated molecular patterns (DAMPs)^4^ and production of inflammatory mediators by dying cancer cells^5,6^ can boost cancer-restraining immune responses. Certain modalities of cell death can drive tumor-specific T cell responses and growth control through exposure of cancer cell-associated target antigens and stimulation of antigen presenting cells^2,5–7^. Moreover, some cytotoxic agents, often referred to as immunogenic cell death (ICD) inducers, have been shown to be more efficient than others at promoting immune-mediated control^3^. Accordingly, various studies exposed the benefit of combining immune checkpoint inhibitors and cytotoxic therapy^8–10^ and numerous clinical trials are currently evaluating these combinations across cancer types^11^. In non-small cell lung cancer (NSCLC)^12,13^, urothelial cancer^14^ and triple negative breast cancer (TNBC)^15–18^, among other cancer types, combinations of CTX and ICB are already approved and used as first-line treatments^11^.

In sharp contrast with these findings supporting immune-mediated benefit of cytotoxic agents, preclinical and clinical data indicate that these treatments can paradoxically have detrimental protumorigenic effects. Various mechanisms have been proposed to explain the latter^19,20^, among which is the Révész effect, a long-appreciated phenomenon by which lethally-irradiated cancer cells stimulate the growth of live cells^21^. Similarly, extensive evidence supports the notion that dead cells can promote immunological tolerance or drive inflammatory responses that fuel tumor progression^22–25^. Definitively, tumor repopulation during cytotoxic therapy regimens and the development of radio or chemoresistance remain a major cause of treatment failure^19,20,26^. These data argue that anti-tumor immune responses during or post-treatment are non-existent, ineffective or overcome by immune evasive mechanisms.

In recent work we have uncovered a dominant role for cancer cell-intrinsic cyclooxygenase (COX)−2 expression and activity of the downstream lipid prostaglandin E_2_ (PGE_2_) in shaping the intratumoral inflammatory milieu and promoting tumor progression through immune escape^27–29^. Furthermore, we have shown that pharmacological inhibition of the COX-2/PGE_2_ axis with anti-inflammatory drugs used at doses considered safe for human use, including selective COX-2 inhibitors, can augment the efficacy of ICB^30^. In other studies, necrotic cell-derived PGE_2_ has been proposed to be an inhibitory DAMP that tempers the immunostimulatory effect of dead cells^31^, and caspase 3-mediated PGE_2_ release by dying cancer cells has been implicated in cancer cell repopulation post-cytotoxic therapy^32,33^. Thus, PGE_2_ release from dying cancer cells has been implicated in contributing to cytotoxic therapy resistance by enhancing tumor cell proliferation and outgrowth.

Based on these findings, we hypothesize that increased PGE_2_ release by dying cancer cells influences the intratumoral inflammatory response after cytotoxic therapy and thus contributes to the conflicting reported outcomes of these mainstream treatments. To test this, we first examine the prevalence of PGE_2_ induction post-cytotoxic therapy across cancer cells of multiple origins and study the mechanistic basis for PGE_2_ induction after CTX. The insight from this analysis allows us to design a real-time live-imaging experimental setup to monitor the growth kinetics of cancer cells alongside induction of the COX-2/PGE_2_ pathway during CTX-treatment. We use this system for a high throughput compound library screen containing 1280 marketed, approved drugs and covering multiple CTX drugs with differing mechanisms of action. To support this, we analyze COX-2 expression changes over time by mining a database of 60 human tumor cell lines treated with various CTX drugs^34^. Finally, we examine the impact of COX-2/PGE_2_ upregulation on the inflammatory features of CTX-treated cells in vivo, and assess the value of pharmacological COX-2 inhibition during the combination of CTX and ICB in murine models, including a poorly immunogenic, spontaneously metastatic TNBC model insensitive to dual chemoimmunotherapy.

## Results

### Cytotoxic therapy induces COX-2-mediated PGE_2_ release from cancer cells naturally expressing COX-2

**Fig. 1:**
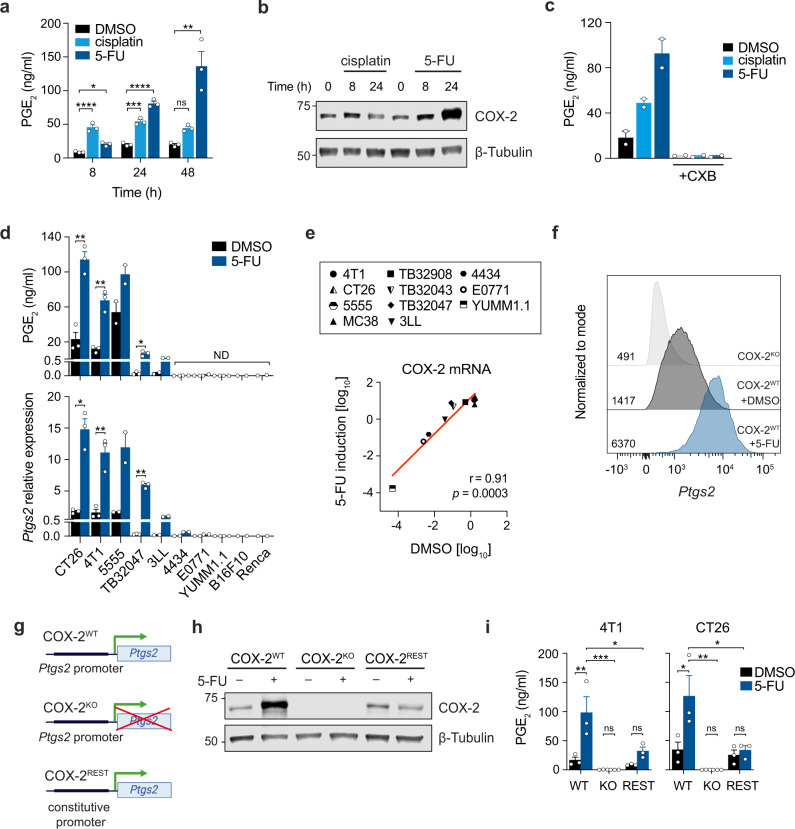
Cytotoxic therapy-induced release of PGE_2_ is exclusive to COX-2 expressing tumor cells and requires transcriptional upregulation of *Ptgs2* via endogenous promoter activity. **a** 4T1 tumor cells were treated with cisplatin (50 µM) or 5-FU (100 µM) and PGE_2_ release into the cell culture medium was measured over time. Mean ±SEM of triplicate wells, representative plot shown of *n* = 2 independent experiments. **b** COX-2 protein levels in 4T1 cells following treatment with cisplatin (50 µM) or 5-FU (100 µM). β-Tubulin ran as a loading control on the same membrane. Data representative of *n* = 2 independent experiments. **c** PGE_2_ release from 4T1 cells treated with cisplatin (20 µM) or 5-FU (100 µM) for 24 h in the presence or absence of the selective COX-2 inhibitor celecoxib (CXB, 5 µM). Mean ±SEM of *n* = 2 independent experiments with triplicate wells. **d** PGE_2_ release (top panel) and *Ptgs2* expression relative to *Hprt* (bottom panel) in multiple murine tumor cell lines following 24 h 5-FU (100 µM) treatment. ND = not detected. Mean ±SEM of *n* = 3 (CT26, 4T1, TB32047), 2 (5555, 3LL, 4434, E0771, YUMM1.1) independent experiments with duplicate wells, or 1 (B16F10, Renca) with duplicate wells. **e** Log10 normalized values of COX-2 transcript expression relative to *Hprt* in tumor cell lines with or without 24 h 5-FU (100 µM) treatment. Spearman’s rank correlation coefficient and *p* value are shown. Murine tumor lines are from multiple origins, including: melanoma (5555, 4434, YUMM1.1, B16F10), colorectal (CT26, MC38), breast (4T1, E0771), lung (3LL), renal (Renca) and pancreatic (TB32043, TB32047, TB32908). *n* = 1 (B16F10, Renca, TB32043, MC38), 2 (5555, 3LL, 4434, E0771, YUMM1.1, TB32908) or 3 (CT26, 4T1, TB32047) independent experiments with duplicate wells. **f** Histograms showing the expression of *Ptgs2* in COX-2^KO^ 4T1 cells and in DMSO- or 5-FU- (100 µM) treated COX-2^WT^ 4T1 cells at 24 h determined using PrimeFlow by gating on live, *Gapdh*^*+*^ cells. Geometric mean fluorescence intensity per group is shown. **g** Schematic depicting promoter control of *Ptgs2* expression in COX-2^WT^, COX-2^KO^ and COX-2^REST^ cells. **h** COX-2 protein levels in 4T1 cells following 48 h treatment with 5-FU (100 µM). β-Tubulin ran as a loading control on the same membrane. Data representative of *n* = 2 independent experiments. **i** PGE_2_ release from 4T1 and CT26 cells treated with 5-FU (100 µM) for 24 h. Mean ±SEM of *n* = 3 independent experiments with duplicate wells. ns = not significant, *p < 0.05, **p < 0.01, ***p < 0.001, ****p < 0.0001 as determined by one-way ANOVA with Tukey’s multiple comparisons test (**a**, **i**) or unpaired two-tailed t-test (**d**). Source data and exact *p* values are provided as a Source Data file.

To test whether and how cytotoxic therapy increases the release of PGE_2_ from cancer cells and the kinetics of this phenomenon, we treated 4T1 breast cancer cells, previously shown to release PGE_2_ after radiotherapy^32^, with the widely used CTX drugs cisplatin or 5-fluorouracil (5-FU). Both drugs led to a substantial increase in the levels of PGE_2_ in the culture medium compared with DMSO-treated control cells (Fig. 1a). Whilst PGE_2_ reached its maximum levels at 8 h post-cisplatin treatment, it continued to rise following 5-FU treatment reaching a seven-fold increase at 48 h compared with control cells. In line with the kinetics of PGE_2_ release, COX-2 protein levels showed a similar kinetic of upregulation (Fig. 1b). The selective COX-2 inhibitor celecoxib (CXB) blunted PGE_2_ synthesis from both control and CTX-treated cells (Fig. 1c), indicating a major contribution for COX-2 and not COX-1 in PGE_2_ induction following treatment. Irradiation of cells with ionizing X-rays or UV light also promoted a marked increase in PGE_2_ release over time (Supplementary Fig. 1a, b).

Next, we examined how prevalent PGE_2_ and COX-2 upregulation was post-CTX treatment across multiple murine cancer cell lines of different tissue origins, covering breast, colorectal, melanoma, pancreatic, lung and renal cancer. PGE_2_ induction following CTX was a common phenomenon which was accompanied by a marked increase in *Ptgs2* (the gene encoding for COX-2) mRNA levels (Fig. 1d). Interestingly, however, the rise in PGE_2_ occurred exclusively in cancer cells that have detectable baseline levels of PGE_2_ and *Ptgs2* (Fig. 1d). Notably, the magnitude of *Ptgs2* induction post-treatment was strictly proportional to *Ptgs2* baseline expression levels across all cell lines tested (Fig. 1d, e). All cells treated with CTX ultimately died following treatment, becoming apoptotic and then secondary necrotic as revealed by monitoring caspase-3/-7 proteolytic activity and membrane permeability using propidium iodide (PI), respectively (Supplementary Fig. 2a, b). Together, these data establish activation of the COX-2/PGE_2_ axis post-cytotoxic therapy as a widespread phenomenon that uniformly occurs in cancer cells, but only in those with prior activation of the pathway.

### The rise in PGE_2_ production following CTX is strictly dependent on transcriptional upregulation of *Ptgs2*

Given that CTX-driven COX-2 upregulation and PGE_2_ release occurred only in cells with baseline *Ptgs2* expression (Fig. 1d), we reasoned that treatment-driven PGE_2_ induction might depend on de novo *Ptgs2* transcription. To test this, we compared the effect of 5-FU treatment in 4T1 parental COX-2-expressing (COX-2^WT^) cells, CRISPR-generated COX-2-deficient (COX-2^KO^) cells or COX-2-deficient cells with restored COX-2 expression (COX-2^REST^) (Fig. 1g). In the latter, COX-2 expression is driven by an unrelated constitutive promoter. Hence, if PGE_2_ enhancement relies on increased *Ptgs2* transcription via specific activation of its promoter regulatory region, it should not occur in COX-2^REST^ 4T1 cells. In agreement with this hypothesis, 5-FU failed to upregulate COX-2 protein and PGE_2_ in COX-2^REST^ cells, while basal levels were comparable between untreated COX-2^WT^ and COX-2^REST^ cells (Fig. 1h, i). The dependence on endogenous COX-2 promoter activity for PGE_2_ induction was also validated in CT26 colorectal COX-2^REST^ cells (Fig. 1i). Altogether, these data formally established specific transcriptional activation of the COX-2 gene as the underlying mechanism responsible for the elevated PGE_2_ production in CTX-treated cancer cells.

### Caspase activity, ROS production, NF-κB and C/EBPβ signaling contribute to, but are dispensable for, COX-2/PGE_2_ pathway upregulation post-CTX

**Fig. 2:**
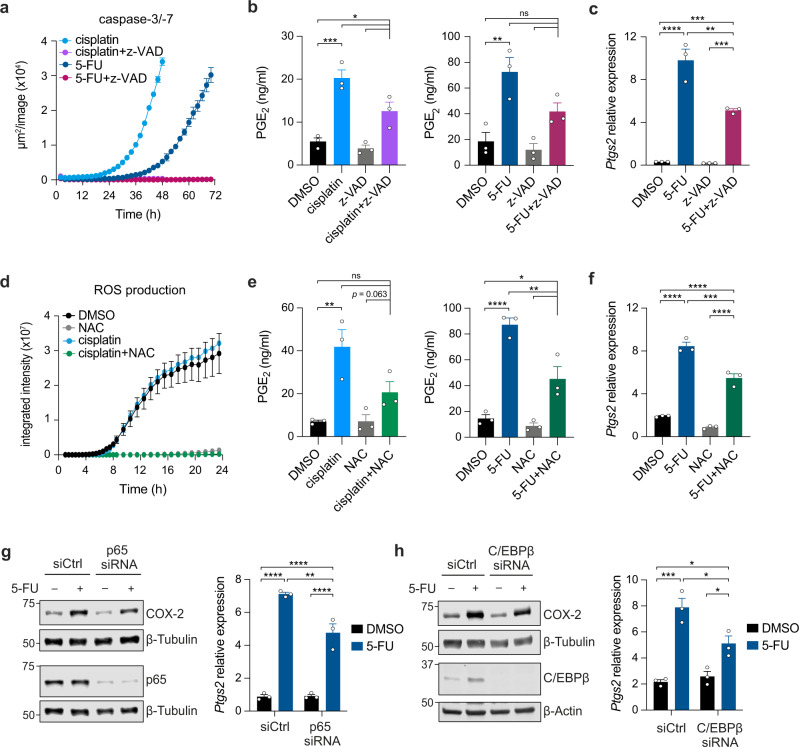
Chemotherapy-induced PGE_2_ release occurs prior to caspase-3/-7 activation and caspase, ROS, NF-κB and C/EBPβ signaling contribute to chemotherapy-driven *Ptgs2* transcription. **a** Caspase-3/-7 activation in 4T1 cells treated with cisplatin (50 µM) or 5-FU (100 µM) in the presence or absence of pan-caspase inhibitor z-VAD-FMK (z-VAD, 100 µM). Mean ±SEM of duplicate wells, representative plot shown of *n* = 3 independent experiments. **b** PGE_2_ release from 4T1 cells treated with cisplatin (50 µM) for 8 h (left panel) or 5-FU (100 µM) for 24 h (right panel) in the presence or absence of z-VAD. Mean ±SEM of *n* = 3 independent experiments with duplicate wells. **c** *Ptgs2* expression relative to *Hprt* in 4T1 cells following 24 h 5-FU (100 µM) and/or z-VAD (100 µM) treatment. Mean ±SEM of triplicate wells, representative plot shown of *n* = 2 independent experiments. **d** Reactive oxygen species (ROS) produced by 4T1 cells treated with cisplatin (50 µM) in the presence or absence of ROS scavenger N-acetyl-L-cysteine (NAC; 5 mM). Mean ±SEM of duplicate wells. **e** PGE_2_ release from 4T1 cells treated with cisplatin (50 µM) for 8 h (left panel) or 5-FU (100 µM) for 24 h (right panel) in the presence or absence of NAC (5 mM). Mean ±SEM of *n* = 3 independent experiments with duplicate wells. **f** *Ptgs2* expression relative to *Hprt* in 4T1 cells following 24 h 5-FU (100 µM) and/or NAC (5 mM) treatment. Mean ±SEM of triplicate wells, representative plot shown of *n* = 2 independent experiments. **g**, **h** COX-2 mRNA (relative to *Hprt*) and protein levels in 4T1 cells following 24 h 5-FU (100 µM) treatment. Cells were transfected with control or siRNA targeting p65 (**g**) or C/EBPβ (**h**) 24 h prior to 5-FU treatment. Mean ±SEM representative of triplicate wells, representative plot and westerns shown of *n* = 2 independent experiments. β-Tubulin or β-Actin loading controls from the same membrane are shown. ns = not significant, *p < 0.05, **p < 0.01, ***p < 0.001, ****p < 0.0001 as determined by one-way ANOVA with Tukey’s multiple comparisons test. Source data and exact *p* values are provided as a Source Data file.

Enhanced PGE_2_ release by dying cells post-cytotoxic therapy has been attributed to caspase-3-mediated activation of calcium-independent phospholipase A_2_^32^. In our experimental system, caspase-3/-7 activity was detected at least 12 h later than the peak in PGE_2_ release from cisplatin and 5-FU-treated cells (Fig. 1a, Fig. 2a, Supplementary Fig. 2a, b), suggesting CTX-driven PGE_2_ upregulation can occur in a caspase-3-independent manner. To explore this further, we used the pan-caspase inhibitor z-VAD-FMK, which effectively inhibited caspase-3/–7 activity in CTX-treated cells without preventing cancer cell arrest and eventual death, as determined by PI staining (Fig. 2a, Supplementary Fig. 2a, b). Whilst z-VAD-FMK decreased the levels of PGE_2_ levels measured in the supernatant from cisplatin- or 5-FU-treated cells, the magnitude of induction was not significantly altered (Fig. 2b, Supplementary Fig. 3a). Similarly, 5-FU-induced COX-2 transcript levels were reduced in the presence of caspase inhibition but a marked induction was still observed from z-VAD-FMK-treated control cells (Fig. 2c).

Reactive oxygen species (ROS), typically produced by stressed cells, have also been implicated in COX-2 upregulation^35,36^. To test their involvement, we treated cancer cells with cisplatin or 5-FU in the presence of the ROS scavenger N-acetyl-L-cysteine (NAC). Addition of NAC fully impaired ROS accumulation and led to a partial but significant decrease in PGE_2_ production with either cisplatin or 5-FU treatment (Fig. 2d, e). An analogous effect was seen for CTX-induced transcriptional upregulation of *Ptgs2* (Fig. 2f). However, no clear decrease in the fold change of PGE_2_ or *Ptgs2* induction was observed (Fig. 2f, Supplementary Fig. 3b), indicating that, like caspase activity, ROS contribute to, but are not essential for COX-2/PGE_2_ pathway induction post-CTX treatment.

We next examined the role of transcription factors predicted to bind to the *Ptgs2* promoter region and previously linked to COX-2 expression and upregulation in different experimental settings^37–39^. First, we tested nuclear factor kappa B (NF-κB), often associated with COX-2 induction including downstream of caspase-3 activity^37,40^. siRNA-mediated knockdown of the key NF-κB subunit p65 led to reduced 5-FU-driven COX-2 protein and mRNA upregulation, but did not noticeably alter the rise in PGE_2_ levels (Fig. 2g, Supplementary Fig. 3c). p65 knockdown had a pronounced effect on *Il6* induction post-5-FU treatment, arguing against incomplete p65 silencing for the partial reduction in *Ptgs2* upregulation (Supplementary Fig. 3d). Similar to NF-κB, knockdown of C/EBPβ, but not of Sp1 or the AP-1 subunit c-Jun, also slightly blunted the increase in COX-2 protein and mRNA post-5-FU, whilst PGE_2_ levels remained unaffected (Fig. 2h, Supplementary Fig. 3c, e, f). Together, these data indicate that NF-κB and C/EBPβ, but not Sp1 or Ap-1, strengthen the upregulation of COX-2 following CTX treatment. However, the induction was still pronounced in their absence suggesting potential redundancy or the activity of a yet to be identified pathway.

### All classes of CTX drugs induce *Ptgs2* upregulation whilst concomitantly arresting cancer cell proliferation

**Fig. 3:**
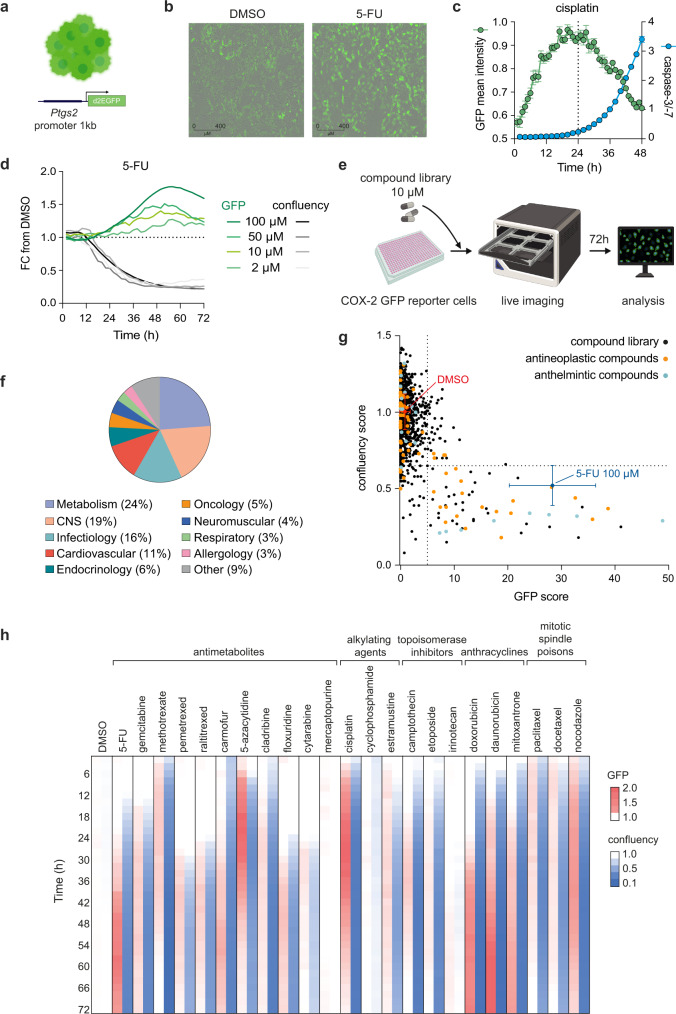
Compound library screen reveals all classes of chemotherapy drugs induce *Ptgs2* expression whilst concomitantly arresting tumor cell proliferation. **a** Schematic of 4T1 COX-2 GFP reporter cells expressing destabilized GFP (d2EGFP) under the control of 1 kb endogenous *Ptgs2* promoter. **b** Representative images of GFP reporter cells treated with DMSO or 5-FU (100 µM) for 24 h. Images are representative of triplicate wells with one field of view per well. **c** Mean intensity of GFP in 4T1 COX-2 reporter cells and caspase-3/-7 activation in 4T1 cells treated with cisplatin (50 µM) over time. Mean ±SEM of duplicate wells. **d** Fold change in GFP mean intensity and tumor cell confluency from DMSO-treated controls in reporter cells treated with different concentrations of 5-FU. **e** Schematic showing the workflow for the compound library screen. **f** Composition of the compound library by therapeutic category. **g** Dot plot showing GFP scores (area under the curve of the fold change in mean fluorescence intensity from DMSO controls over 72 h) and confluency scores (mean of the fold change in confluency from DMSO controls over 72 h) for each compound within the library (1280 in total). Each dot represents one compound in the library, drugs denoted as having antineoplastic therapeutic effects are highlighted in orange and anthelmintic effects in light blue. DMSO and 5-FU controls are shown as mean ± SD of all replicate wells across multiple plates ran in the screen. Dashed lines indicate GFP score of 5 and confluency score of 0.65. **h** Heatmap depicting fold change in GFP mean fluorescence intensity and confluency compared with DMSO controls over time for different chemotherapy drugs of various classes within the compound library screen. All compounds were tested at 10 µM, except for 5-FU (100 µM) and cisplatin (50 µM). Source data are provided as a Source Data file.

We next sought to investigate in closer detail the kinetics of *Ptgs2* transcriptional upregulation post-CTX relative to the treatment effects on cancer cell growth. To this aim, we generated COX-2 transcription reporter cells stably expressing destabilized GFP (d2EGFP) under the control of the endogenous COX-2 promoter, such that we could concomitantly monitor *Ptgs2* upregulation alongside cancer cell growth (Fig. 3a). We used a region of the *Ptgs2* promoter spanning about one kilobase (kb) upstream of the transcription start site which contains the key regulatory elements that control *Ptgs2* transcription^37,41^. 4T1 cells retrovirally transduced with this construct expressed GFP, and showed a dramatic increase in GFP fluorescence intensity post-CTX treatment (Fig. 3b, c, Supplementary Fig. 4a), whereby the GFP mean intensity at a particular time was calculated by averaging across all GFP^+^ cells in a given well (see Methods). Live-imaging of these cells treated with cisplatin showed a steady increase in GFP fluorescence intensity over time, peaking around 24 h and subsequently decreasing (Fig. 3c). The decay in GFP signal coincided with the detection of caspase-3/-7 proteolytic activity, and continued waning whilst the cells became macroscopically apoptotic and eventually secondary necrotic (Fig. 3c, Supplementary Fig. 4b). Titrating doses of 5-FU revealed a clear time- and dose-dependent increase in GFP signal which peaked at 48 h, one day later than in cisplatin-treated cells. These differences in GFP induction are in agreement with the different kinetics of *Ptgs2* and PGE_2_ upregulation induced by these two CTX drugs (Fig. 3c, d, Supplementary Fig. 4a–c). We simultaneously measured GFP and endogenous *Ptgs2* mRNAs in the COX-2 reporter cells using quantitative PCR, which further indicated comparable induction of both transcripts post-CTX (Supplementary Fig. 4d). Together, these data validated the use of this cell line as a faithful reporter of endogenous COX-2 transcriptional activity post-CTX, with the kinetics of GFP upregulation closely reflecting those of *Ptgs2* (Supplementary Fig. 4a–c).

We exploited this reporter cell line and devised a high throughput system to screen a comprehensive library of 1280 market-approved compounds and simultaneously compare their effects on cancer cell proliferation and *Ptgs2* transcription over time (Fig. 3e). The library comprised compounds approved for use in a diverse range of therapeutic applications (Fig. 3f) with antibacterial (13%), anti-inflammatory (8%), antihypertensive (8%), antineoplastic (5%) and analgesic (5%) drugs being the top 5 represented groups. Critically, it contained multiple CTX drugs with different mechanisms of action covering alkylating agents, anti-metabolites, topoisomerase inhibitors, mitotic spindle poisons and anthracyclines (Supplementary Table 1). Using the IncuCyte platform, we monitored cell confluence and GFP expression by real-time live-imaging for a period of 72 h (Fig. 3e). To compare the effect of the multiple library compounds, we calculated cell confluence scores by averaging the fold change in confluence over the whole culture time relative to DMSO-treated controls ran in each individual 384-well plate (see Methods). We also generated GFP scores by calculating the area under the curve (AUC) of the fold change in GFP fluorescence intensity for each compound relative to DMSO-treated controls over 72 h. This analysis showed that drugs that resulted in a noticeable increase in GFP fluorescence (GFP score >5) also greatly reduced cell growth (confluency score <0.65) (Fig. 3g). In particular, from the total 69 *bona* *fide* antineoplastic drugs tested, all compounds that diminished 4T1 cell proliferation also increased GFP. Indeed, there was a striking anti-correlation between cell confluency and GFP scores for this subgroup of drugs (Spearman r = −0.70, *p* < 0.0001), irrespective of their varied mechanisms of action. Anthelmintics, another group of compounds highly represented in this category (confluency score <0.65, GFP score >5) and with reported antineoplastic effects^42,43^, similarly induced GFP whilst concomitantly restricting tumor cell growth (Spearman r = −0.73, *p* = 0.0001). Examination of cell growth and GFP fluorescence kinetics over time indicated that GFP induction largely coincided with the point at which exponential cell growth plateaued and the cells started dying (Fig. 3h, Supplementary Fig. 4e). Of note, anthracyclines, prototypical inducers of ICD, and non-ICD inducing agents, such as gemcitabine, equally showed this pattern. Therefore, high throughput screening of a large market-approved compound library on COX-2-reporter 4T1 cells revealed that all classes of CTX drugs drive *Ptgs2* upregulation alongside inhibiting tumor cell proliferation.

### CTX universally increases COX-2 mRNA levels in human cells with basal COX-2 expression

**Fig. 4:**
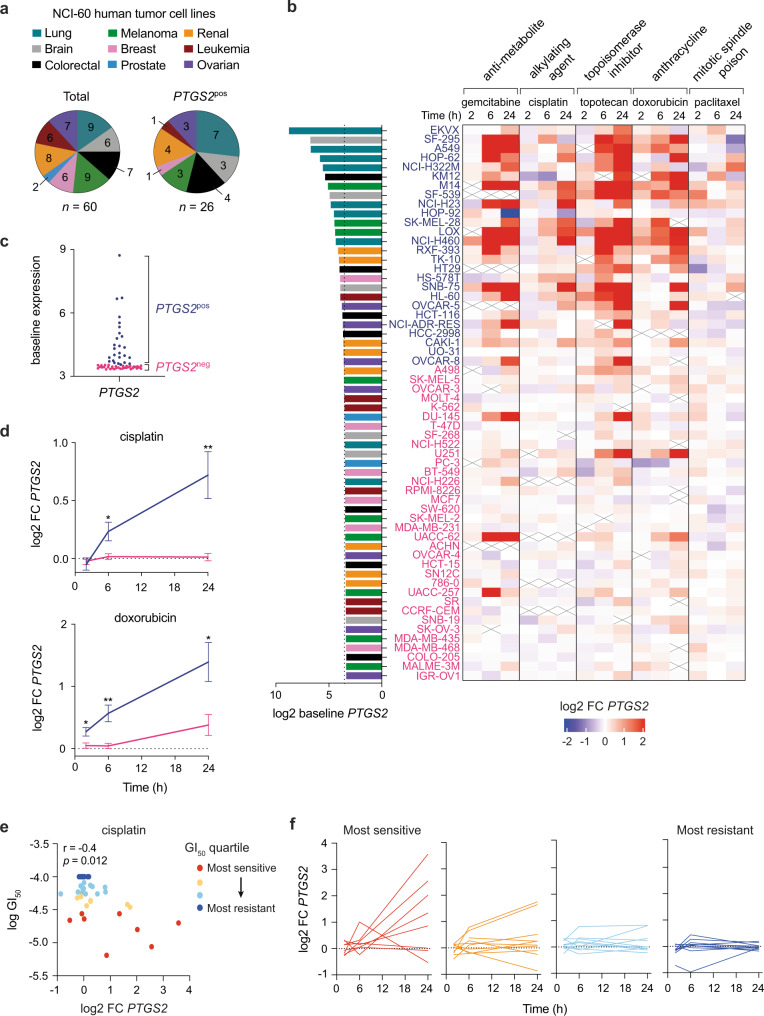
Chemotherapy drugs with different mechanisms of action increase COX-2 mRNA levels in human cancer cells with high basal COX-2 expression. **a** Composition of the total NCI-60 panel of human cancer cell lines by tumor type (left) and frequency of tumor types defined as *PTGS2*^*pos*^ (right). **b** Heatmap showing log2 fold change in *PTGS2* expression in 60 human tumor cell lines treated with different chemotherapy drugs for 2 h, 6 h or 24 h. Cell lines are ranked from highest to lowest *PTGS2* expression at baseline, those defined as *PTGS2*^pos^ are shown in blue and *PTGS2*^neg^ in pink. A cross is shown where data were not available. **c** Baseline expression of *PTGS2* per cell line, segregated into *PTGS2*^pos^ (*n* = 26) or *PTGS2*^neg^ (*n* = 34). **d** Log2 fold change in *PTGS2* expression over time for *PTGS2*^pos^ or *PTGS2*^neg^ cell lines treated with cisplatin or doxorubicin. *p < 0.05, **p < 0.01 as determined by mixed-effects analysis with Sidak’s multiple comparisons test. **e** Dot plot showing log2 fold change in *PTGS2* at 24 h against log10 GI_50_ values for cisplatin. Cell lines with available GI_50_ data (*n* = 38) were separated into quartiles with the most sensitive (red) to most resistant (dark blue) shown. Spearman’s rank correlation coefficient and *p* value is shown. **f** Log2 fold change in *PTGS2* values over time for cell lines treated with cisplatin, grouped based on GI_50_ quartile. Most sensitive Q1 (red, *n* = 8), Q2 (orange, *n* = 10), Q3 (light blue, *n* = 10), most resistant Q4 (dark blue, *n* = 10). Log2 fold change and GI_50_ data were downloaded from the NCI Transcriptional Pharmacodynamics Workbench^34^. Source data and exact *p* values are provided as a Source Data file.

We next evaluated if upregulation of COX-2 transcription also occurred in human cancer cells and was similarly independent of the class of CTX drug. For this, we interrogated a dataset of the NCI-60 human cancer cell line panel, in which cancer cells from nine different tumor types (Fig. 4a) were treated with diverse anti-cancer drugs and the transcriptome analyzed over time^34^. CTX drugs, irrespectively of their mechanism of action and tissue of origin, raised *PTGS2* transcript levels in a time-dependent manner in approximately half of all cell lines tested (Fig. 4b). We investigated whether this upregulation was dependent on baseline *PTGS2* expression, as described above in murine cancer cells. For this, we separated the NCI-60 panel into *PTGS2* positive (*PTGS2*^*pos*^, *n* = 26) and negative (*PTGS2*^*neg*^, *n* = 34) according to their basal expression levels (Fig. 4c). In line with the mouse data, the vast majority of *PTGS2*^*pos*^ but only a few *PTGS2*^*neg*^ cancer cell lines upregulated COX-2 transcription post-CTX treatment (Fig. 4b). Likewise, a prototypical ICD-inducer, doxorubicin, and a non-ICD drug, cisplatin, similarly induced *PTGS2* (Fig. 4d). We also validated CTX-induced PGE_2_ release in a *PTGS2* positive human cell line but did not detect PGE_2_ from a *PTGS2* negative line (Supplementary Fig. 5).

We further explored whether there was an association with responsivity to treatment, given the observed relationship with COX-2 upregulation and proliferation arrest in mouse cancer cells. For 3/5 of the CTX drugs, there was a significant inverse correlation between the sensitivity of the cancer cells to the drug (expressed as the mean 50% growth inhibitory concentration, GI_50_) and *PTGS2* induction (Fig. 4e, f, Supplementary Fig. 6a, b). More sensitive cancer cell lines, with a lower GI_50_, were those which typically displayed enhanced *PTGS2* expression after treatment. Therefore, COX-2/PGE_2_ axis activation by CTX-treated cancer cells is a prevalent phenomenon conserved in mice and humans, which occurs irrespective of the tissue of origin and antineoplastic agent used, but is reliant on baseline cancer cell expression of COX-2 and sensitivity to drug treatment.

### CTX-induced COX-2 upregulation modifies the inflammatory response to dying tumor cells in vivo

**Fig. 5:**
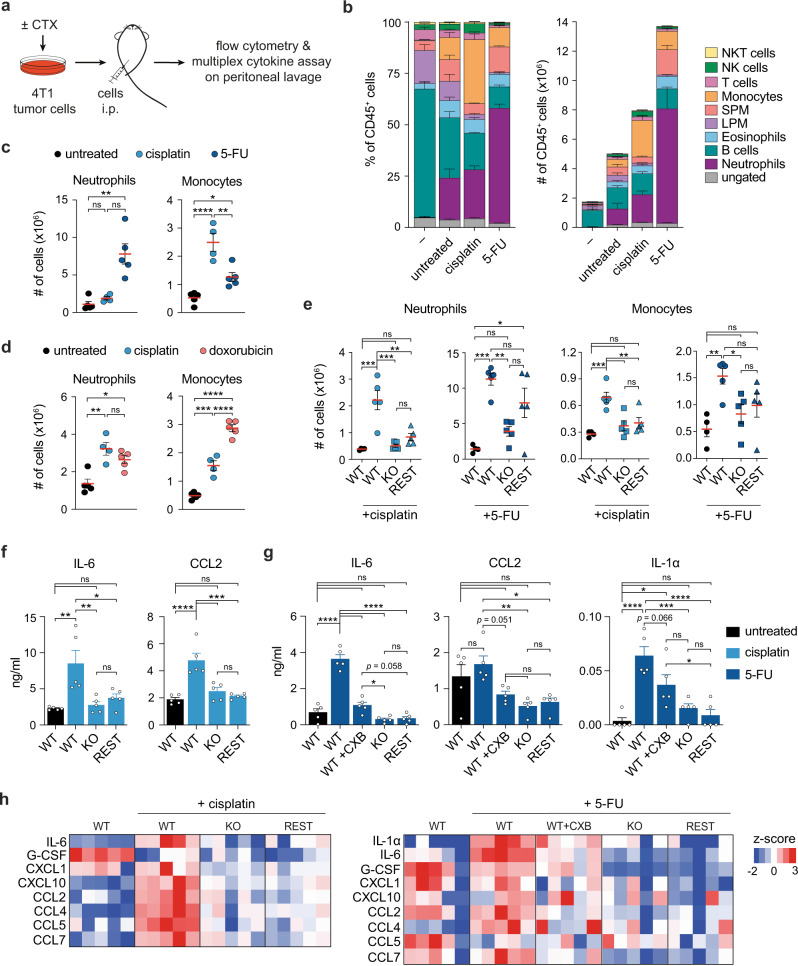
Chemotherapy-induced COX-2 expression modulates the inflammatory response to dying tumor cells. **a** 4T1 tumor cells were pre-treated with cisplatin (50 µM) or doxorubicin (10 µM) for 4 h or 5-FU (100 µM) for 24 h. Cells were injected intraperitoneally (i.p.) and the next day the peritoneal lavage was collected for analysis. **b** Frequency (left panel) and total number (right panel) of live CD45^+^ immune cells present in mice injected with PBS (-), untreated, cisplatin or 5-FU treated 4T1 cells. LPM = large peritoneal macrophages, SPM = small peritoneal macrophages. *n* = 4 (cisplatin) or 5 (PBS, untreated, 5-FU) mice per group. **c**, **d** Total number of neutrophils and monocytes recruited by i.p. injection of untreated or CTX-treated 4T1 cells. *n* = 4 (cisplatin) or 5 (untreated, 5-FU, doxorubicin) mice per group. **e** Total number of neutrophils and monocytes recruited by i.p. injection of untreated or CTX-treated 4T1 COX-2^WT^, COX-2^KO^, or COX-2^REST^ cells. *n* = 4 (untreated COX-2^WT^) or 5 (all other groups) mice per group. **f**, **g** Concentrations of soluble factors measured within the peritoneal lavage in mice injected with untreated, cisplatin treated cells (**f**) or 5-FU treated cells (**g**). WT + CXB group received cells pre-treated with 5-FU in the presence of the COX-2 inhibitor celecoxib (CXB, 5 µM). *n* = 5 mice per group. **h** Heatmaps showing detectable soluble factors within the peritoneal lavage. Rows represent z-score normalized ng/ml values, each column represents one mouse, *n* = 5 mice per group. Data in **b**–**g** are represented as mean ± SEM of individual mice, ns = not significant, *p < 0.05, **p < 0.01, ***p < 0.001, ****p < 0.0001 as determined by one-way ANOVA with Tukey’s multiple comparisons test or Kruskal–Wallis test with Dunn’s multiple comparisons test for non-parametric data. Source data and exact *p* values are provided as a Source Data file.

Cancer cell-derived PGE_2_ is a major regulator of the intratumoral inflammatory response^27–29^. Thus, we next sought to determine the impact of increased COX-2/PGE_2_ activity on the inflammatory features of CTX-treated cancer cells in vivo. In order to specifically dissect the inflammatory potential of CTX-treated cancer cells without confounding effects of CTX treatment on non-tumor cells, we modified a well-described experimental system in which cells are injected into the peritoneal cavity of wild-type animals to study the acute inflammatory response to necrotic cells^44,45^. The inflammatory features of the injected cells can then be examined by monitoring their ability to recruit immune cells or measuring the levels of cytokines and chemokines in the peritoneal cavity (Fig. 5a, Supplementary Fig. 7). CTX pre-treatment profoundly altered the proportion and absolute number of immune cells recruited 18 h post-injection when compared with PBS-injected mice or mice receiving an equal number of untreated live 4T1 cells (Fig. 5b–d). Both cisplatin- and 5-FU-treated cells promoted the accumulation of neutrophils and/or monocytes, but to dissimilar degrees consistent with the different kinetics of cell death and COX-2/PGE_2_ upregulation induced by these two drugs (Fig. 1a, b, 2a, Supplementary Fig. 2a, b). The number of other immune cell subsets, including peritoneum-resident leukocyte subsets like large peritoneal macrophages (LPM) and B cells were not noticeably increased compared with mice receiving untreated live 4T1 cells (Fig. 5b).

We next compared the effect of the ICD-inducing agent doxorubicin with that of cisplatin, a non-ICD inducer. Both drugs significantly and similarly increased the recruitment of neutrophils and monocytes compared with untreated 4T1 cells (Fig. 5d, Supplementary Fig. 8). Together, these data suggested that the acute inflammatory features of cancer cells are profoundly altered by CTX treatment. Specifically, treatment with either ICD or non-ICD inducing CTX drugs stimulated a rapid accumulation of myeloid cells, including both neutrophils and monocytes, from the bloodstream.

To assess the contribution of the COX-2/PGE_2_ axis to the inflammatory phenotype of CTX-treated cells, we compared the effect of injecting cisplatin- or 5-FU-treated COX-2^WT^ and COX-2^KO^ 4T1 cells. Remarkably, CTX-treated COX-2^KO^ cells attracted far fewer neutrophils or monocytes than their parental COX-2-expressing counterpart, similar to untreated 4T1 cells (Fig. 5e), exposing a major contribution of cancer cell-intrinsic COX-2 activity. To determine whether the heightened inflammatory properties of CTX-treated 4T1 cells resulted from enhanced COX-2/PGE_2_ activity selectively post-treatment, we used COX-2^REST^ 4T1 cells, which have the pathway constitutively active but do not upregulate it post-CTX treatment (Fig. 1h, i). Pre-treated with either cisplatin or 5-FU, COX-2^REST^ cells were less potent than COX-2^WT^ cells at recruiting neutrophils and monocytes and largely phenocopied CTX-treated COX-2^KO^ cells (Fig. 5e). Together, these results indicated that transcriptional upregulation of COX-2 by cancer cells after CTX, and not their basal expression, largely accounts for their ability to stimulate inflammatory myeloid cell recruitment.

Using the same experimental setup, we next determined the expression levels of multiple cytokines, chemokines and growth factors in the peritoneal lavage (Fig. 5a). In keeping with the changes in leukocyte composition, we found that the levels of IL-6 were markedly and selectively increased following the injection of cisplatin- or 5-FU-treated COX-2^WT^ cells relative to untreated COX-2^WT^, CTX-treated COX-2^KO^ or COX-2^REST^ 4T1 cells (Fig. 5f-h). A similar effect was noticed for CCL2, CCL4, CCL5, CCL7 and CXCL10 with cisplatin-treated cells and IL-1α with 5-FU-treated cells. CXCL1, a major neutrophil chemoattractant, and other inflammatory mediators were detected in the peritoneal cavity, however their levels were either not changed or only moderately different between groups (Fig. 5h). CCL3, CXCL2, CXCL5, CXCL9, GM-CSF, IL-1β, IL-10, IL-12p40, IFNα, IFNγ, TNF and VEGF-A were also measured but undetectable within the lavage. Finally, addition of CXB during pre-treatment of COX-2^WT^ 4T1 cells with 5-FU reduced the levels of IL-6 and IL-1α detected in the peritoneum (Fig. 5g, h), further exposing the major contribution of COX-2 enzymatic activity for the inflammatory properties of CTX-treated cancer cells in vivo. We conclude that transcriptional upregulation of COX-2 and subsequent PGE_2_ release following CTX-treatment is a key determinant of the cellular and molecular features underpinning the inflammatory response induced by dying cancer cells in vivo.

### Co-administration of a COX-2 inhibitor is essential to drive tumor control during ICB and CTX combination therapy

**Fig. 6:**
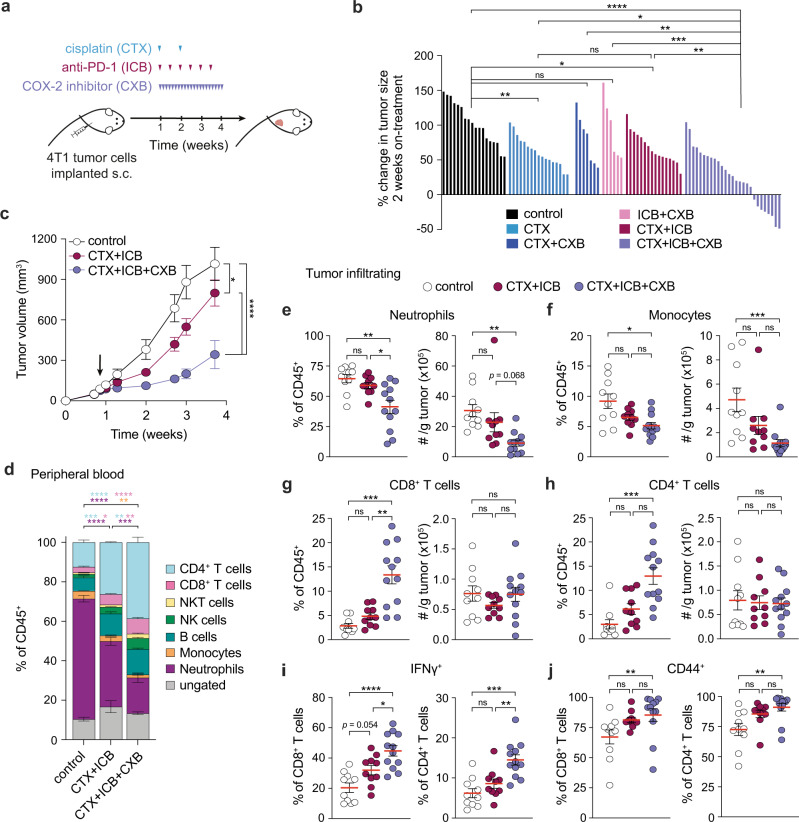
COX-2 inhibition is essential for tumor control during chemotherapy and immunotherapy combination treatment. **a** Mice were inoculated subcutaneously with 4T1 tumor cells and treatment began on day 5-6 post-injection when tumor volumes were 94.0 ± 4.6 mm^3^ (mean ± SEM). **b** Waterfall plot showing percent change in tumor size two weeks post-treatment start, each bar represents one mouse (*n* = 6 (ICB + CXB), 7 (CTX + CXB), 16 (CTX + ICB), 18 (control and CTX) or 27 (CTX + ICB + CXB) per group, pool of four independent experiments). **c** Growth profiles of tumors in mice treated with control (*n* = 10), CTX + ICB (*n* = 10) and CTX + ICB + CXB (*n* = 12), pool of two independent experiments. Arrow indicates treatment start, mice received CXB or vehicle treatment bidaily. **d** Analysis of circulating leukocytes in peripheral blood two weeks on-treatment in mice treated with control (*n* = 5), CTX + ICB (*n* = 5) and CTX + ICB + CXB (*n* = 7), representative plot shown of two independent experiments. Statistical significance for neutrophils, monocytes, CD8^+^ and CD4^+^ T cells is shown. **e**–**j** Analysis of tumor-infiltrating leukocytes three weeks on-treatment in mice treated with control (*n* = 10), CTX + ICB (*n* = 10) and CTX + ICB + CXB (*n* = 12), pool of two independent experiments. Frequency and absolute number normalized per gram of tumor for neutrophils (**e**), monocytes (**f**), CD8^+^ (**g**) and CD4^+^ T cells (**h**). Frequency of IFNγ^+^ (**i**) and CD44^+^ (**j**) of CD8^+^ and CD4^+^ T cells. Data in **c**–**j** are represented as mean ± SEM, ns = not significant, *p < 0.05, **p < 0.01, ***p < 0.001, ****p < 0.0001 as determined by one-way ANOVA with Tukey’s multiple comparisons test or Kruskal–Wallis test with Dunn’s multiple comparisons test for non-parametric data (**b**, **e**-**j**) or two-way ANOVA (**c**). Source data and exact *p* values are provided as a Source Data file.

The above results, alongside our recent findings showing that oral administration of CXB can improve the efficacy of ICB^30^, prompted us to evaluate if pharmacological COX-2 inhibition would improve the efficacy of CTX and ICB combinations. To this aim, we tested the effect of systemic CTX and ICB treatment, using cisplatin and PD-1 blockade, with or without daily oral CXB treatment in mice bearing 4T1 tumors (Fig. 6a). These tumors are very poorly immunogenic and typically unresponsive to cytotoxic therapy or ICB^30,46,47^. Accordingly, cisplatin monotherapy led to a modest delay in tumor growth compared with control-treated mice, with no further benefit derived from the addition of PD-1 blockade (CTX + ICB; Fig. 6b). Dual combinations of CTX and CXB or ICB and CXB failed to induce significant tumor control compared with vehicle-treated mice (Fig. 6b). Crucially, however, triple therapy combining CTX, ICB and COX-2 inhibition uniquely impaired tumor progression, with approximately 30% of mice exhibiting tumor shrinkage two weeks following treatment (Fig. 6b, c).

To test if COX-2 inhibition would enhance the efficacy of chemoimmunotherapy in a different tumor model, using a different CTX drug, we treated CT26 colorectal tumors using 5-FU, commonly used in first-line CTX regimens for the treatment of colorectal cancer (Supplementary Fig. 9a). In line with our recent findings^30^, this tumor model responded vigorously to ICB + CXB, and dual 5-FU and ICB combination also led to a potent response (Supplementary Fig. 9b–d). Yet, mice treated with the triple CTX + ICB + CXB therapy achieved more and faster rejections, with half of the mice having fully rejected their tumor two-weeks post-treatment start (Supplementary Fig. 9b–d). Together, these data demonstrated that concomitant COX-2 inhibition enhances the response to chemoimmunotherapy regimens in models sensitive or refractory to the dual combination.

We next analyzed the effect of treatment on the composition of circulating and tumor-infiltrating immune cells in 4T1 tumor-bearing mice (Supplementary Fig. 10a-c). In the blood, the frequency of neutrophils was lower in CTX + ICB + CXB-treated mice relative to vehicle- or CTX + ICB-treated animals (Fig. 6d). Conversely, the percentage of CD4^+^ and CD8^+^ T cells was highest in mice treated with the triple combination (Fig. 6d). In agreement with these systemic effects, the frequency and number of tumor-infiltrating neutrophils and monocytes were reduced in mice receiving the triple therapy (Fig. 6e, f). Additionally, the fraction of intratumoral CD8^+^ and CD4^+^ T cells was higher in CTX + ICB + CXB-treated mice compared with CTX + ICB- or control-treated mice, with no clear difference in their number per gram of tumor across any of the groups (Fig. 6g, h). Immune phenotypic analysis of tumor-infiltrating T cells revealed that co-administration of CXB to the combination of CTX and ICB boosted the activation of CD8^+^ and CD4^+^ T cells, which displayed significantly higher production of IFNγ and surface expression of the activation marker CD44 (Fig. 6i, j). This immune-infiltrate analysis is consistent with the impaired tumor growth following the triple therapy being immune-mediated. In line with this, the triple combination failed to induce tumor control in immunodeficient NSG mice (Supplementary Fig. 10d, e). Altogether, these data support a model whereby COX-2 inhibition, by altering the inflammatory properties of CTX-treated cancer cells, limits the recruitment of myeloid cells, favors T cell effector function and thereby immune-mediated tumor control when in combination with both CTX and ICB.

**Fig. 7:**
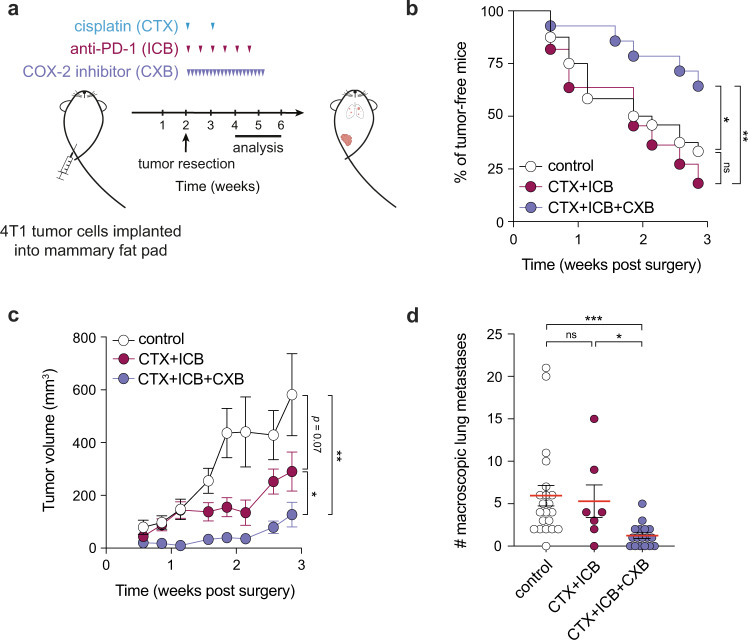
COX-2 inhibition alongside chemotherapy and immunotherapy combination treatment controls tumor relapse and limits metastatic spread in an adjuvant surgery model. **a** Mice were inoculated orthotopically with 4T1 tumor cells, tumors surgically removed two weeks post-inoculation and treatment began the day after surgery. **b** Fraction of tumor-free mice over time (defined as local regrowth at orthotopic site <50 mm^3^). Control (*n* = 24), dual combination (*n* = 11) or triple combination (*n* = 28). **c** Growth profiles of relapsing tumors for mice in (**b**). **d** Number of macroscopic lung nodules in control (*n* = 21), dual combination (*n* = 7) or triple combination (*n* = 16) treated animals analyzed within 30 days of surgery (pool of four independent experiments). Data in (**c**, **d**) are represented as mean ± SEM, ns = not significant, *p < 0.05, **p < 0.01, ***p < 0.001 as determined by Log-rank (Mantel-Cox) test (**b**), two-way ANOVA (**c**) and Kruskal–Wallis test with Dunn’s multiple comparisons test (**d**). Source data and exact *p* values are provided as a Source Data file.

Finally, we devised an experimental system to model adjuvant treatment, as TNBC patients often experience tumor relapse following resection of the primary tumor^18^. We implanted 4T1 cells, considered a TNBC experimental model, orthotopically into the mammary fat pad and two weeks later surgically removed the tumors when they were approximately 300 mm^3^ (Fig. 7a). Using this approach, we monitored the efficacy of chemoimmunotherapy with or without COX-2 inhibition in controlling tumor re-emergence at the primary site and the metastatic spread of 4T1 cells starting treatment post-tumor resection (Fig. 7a). Crucially, the triple combination significantly prevented tumor regrowth at the surgery site, whereas tumors in CTX + ICB treated mice reoccurred at a similar rate to control treated mice (Fig. 7b). Furthermore, the growth of relapsing tumors was greatly impaired only in triple combination-treated mice compared with vehicle-treated animals, with the dual CTX + ICB combo showing lower and delayed efficacy (Fig. 7c). Lastly, both control and CTX + ICB-treated mice exhibited a comparable number of macroscopic lung metastases, significantly higher than mice treated with CTX + ICB + CXB (Fig. 7d). Together, these results suggest that the triple combination of COX-2 inhibition with cytotoxic therapy and immunotherapy can improve outcomes by limiting tumor relapse and metastasis in an adjuvant setting of TNBC, where improvements in therapeutic outcome are urgently required.

## Discussion

Chemotherapy and radiotherapy constitute pillars of oncology treatments and remain mainstream treatment modalities for patients with unresectable cancers. While the value of their direct tumor debulking effects is unquestionable, how cytotoxic therapies influence the inflammatory response and tumor-specific immunity post-treatment is still disputed. Multiple preclinical and clinical studies support a beneficial immune-mediated anti-cancer effect, largely attributed to the induction of ICD, whereby dying or dead tumor cells stimulate T cell-immunity against dead cell-associated antigens^2,3,5–7^. In sharp contrast, abundant evidence exists indicating cytotoxic therapy can paradoxically fuel tumor progression post-treatment, in part through provoking a wound-healing response or pro-tumorigenic cancer inflammation^19,20,22–25^. Given our recent work uncovering a dominant role for cancer cell-intrinsic COX-2/PGE_2_ expression in tumor inflammation and immune evasion^27–29^, we here investigated the impact of the pathway in the inflammatory response induced by cytotoxic therapy-treated cancer cells.

By analyzing multiple cancer lines, we found that enhanced COX-2 expression and PGE_2_ synthesis is an active process that occurs universally across murine and human cancer cells, provided that the COX-2 gene is already being transcribed. High throughput screening of COX-2 transcription using a compound library of 1280 market-approved drugs, combined with quantitative real-time live cell-imaging, revealed that all classes of CTX agents induce *Ptgs2* transcriptional upregulation whilst concomitantly arresting tumor cell growth, independently of their mechanism of action. Curiously, anthelmintic drugs also showed a similar phenomenon, with these compounds reported to induce cell death by interfering with microtubule dynamics^42^, the mechanism of action of mitotic spindle poison CTX drugs such as paclitaxel. These observations suggest there might be a widely conserved mechanism responsible for the acute upregulation of *Ptgs2* transcription, such as early apoptotic mediators, DNA damage response or stress pathways. Indeed, our analysis of human cancer cell lines also indicates that upregulation of COX-2 expression is coupled with sensitivity to drug treatment.

Dissecting the mechanistic basis for the increase in PGE_2_ synthesis in cancer cells during cytotoxic therapy, in contrast to previous reports^32,33,40^, we found the rise in PGE_2_ synthesis preceded caspase-3 proteolytic activity. Moreover, caspase inhibition which has been shown to drive augmented production of inflammatory mediators by dying cells via NF-kB activation^5,6^, did not increase PGE_2_ production. Instead, PGE_2_ levels post-treatment strictly depended on the transcriptional upregulation of *Ptgs2*, and accurately correlated with its basal expression levels in mouse and human cancer cells. Our analysis excluded a requirement for the transcription factors Sp1 and AP-1, but implicated a partial role for NF-kB and C/EBPβ, as well as caspase activity and ROS in strengthening *Ptgs2* induction following CTX treatment. Given how prevalent this phenomenon was across mouse and human cells, and the variety of treatments that could trigger it, we speculate that this response can be induced through multiple distinct pathways acting in concert on the complex regulatory promoter sequence upstream of the COX-2 gene. Indeed, similar to what has been reported for constitutive COX-2 expression across different tissues^39^, our findings highlight the need for a more complete understanding of COX-2 transcriptional regulation in inflammation and cancer.

The compound library screen also uncovered different kinetics of cell growth arrest and apoptotic induction by multiple CTX drugs. Interestingly, the halt in proliferation largely coincided with the point at which *Ptgs2* upregulation began, with no drug in the wide-ranging library greatly inducing *Ptgs2* without diminishing tumor cell proliferation. Crucially, induction of *Ptgs2* transcription happened broadly for cytotoxic treatments irrespective of whether they can trigger ICD. Anthracyclines, prototypical ICD-inducers, upregulated *Ptgs2* transcription similar to drugs not linked to the induction of T cell immunity post-treatment, such as cisplatin or gemcitabine^3^. Of note, a recent study reported that gemcitabine can be converted into an ICD-inducing drug through PGE_2_ blockade^48^. Our data are in line with these results, but argue that activation of the COX-2/PGE_2_ pathway occurs irrespective of the cytotoxic therapy agent used and as long as there is prior pathway activity. This finding has important implications for guiding treatment selection in the clinic, as tumors with low or no basal cancer cell COX-2 expression might not show acute increased COX-2 activity. COX-2 upregulation post-cytotoxic therapy has been reported in NSCLC patients^49^ but requires further investigation across different cancer types. Currently available datasets of tumor specimens from patients treated with conventional CTX or radiotherapy mostly correspond to tumor biopsies or resections obtained sometime after and not during or immediately post-treatment, where we speculate evidence of pathway induction should be examined. Additionally, other cell types within the tumor microenvironment can express high levels of COX-2, such as neutrophils^50^ and fibroblasts^51^. Whether non-tumor cells exposed to cytotoxic therapy also upregulate the COX-2/PGE_2_ axis and influence the inflammatory response warrants further investigation.

By CRISPR-mediated ablation of COX-2 or genetic engineering of its transcriptional activity by swapping the regulatory promoter region controlling *Ptgs2* transcription, we were able to expose the selective and profound impact of augmented COX-2 activity on the inflammatory properties of CTX-treated cancer cells in vivo. Modifying an experimental system^44,45^ to enable interrogation of the effects of CTX drugs on cancer cells without the pleiotropic and confounding effects of CTX on non-tumor cells, we found that treatment-induced upregulation of the COX-2 pathway drives acute recruitment of circulating neutrophils and monocytes. Equally, CTX-treated cancer cells led to a marked increase in inflammatory cytokines and chemokines, which depended on the transcriptional upregulation of COX-2 specifically post-CTX. Pharmacological inhibition of COX-2 enzymatic activity with a selective inhibitor prevented the increase in these soluble factors, confirming the dominance of COX-2 activity for the inflammatory effects of the dying cancer cells. Interestingly, 5-FU-treated cells were consistently more potent at recruiting neutrophils in this experimental system compared with cisplatin treated cells. We observed that 5-FU-treated cells exhibited slower cell death kinetics in vitro, undergoing proliferative arrest for a longer time period with enhanced PGE_2_ release compared with cisplatin-treated cells. We therefore speculate the strength of the inflammatory response depends upon the kinetics of COX-2 upregulation and cell death induced by different cytotoxic agents.

Whether the observed COX-2-mediated inflammatory effects of CTX-treated dying cancer cells promote or hinder CTX efficacy cannot be easily inferred. PGE_2_ can have pleiotropic and often contrasting effects on the immune system^52,53^, recently implicated both in subverting anti-tumor immunity^27–30^ as well as promoting immunosurveillance of senescent cells and suppression of early tumorigenesis^54^. Additionally the contribution of myeloid cells, such as neutrophils, to the efficacy of cytotoxic therapy is also conflicted^55^. Numerous studies have demonstrated protumorigenic effects of neutrophils and roles in driving metastasis^46,50,56–63^. However, there is evidence for beneficial anti-tumor effects^64–68^, including in the context of CTX treatment^69^. Definitively, in the context of ICB, accumulation of myeloid cells at the tumor site is more commonly associated with immune suppression^47^. Thus, to evaluate the potential therapeutic implications of COX-2 upregulation by dying cancer cells for tumor immunity, we determined the impact of pharmacologically inhibiting COX-2 alongside ICB and CTX. This latter dual combination is a promising treatment modality approved as first-line treatment in many malignancies with mixed results^11,70^. In a poorly immunogenic, metastatic TNBC model insensitive to the dual combination of cisplatin and PD-1 blockade, we showed that concomitant COX-2 inhibition is required to promote significant immune-mediated tumor control. Addition of CXB to ICB and CTX was accompanied by diminished accumulation of intratumoral neutrophils and monocytes, and an increase in the fraction of tumor-infiltrating IFNγ-producing CD4^+^ and CD8^+^ T cells. Furthermore, in a model partially responsive to a chemoimmunotherapy regimen, the addition of a COX-2 inhibitor accelerated tumor control and led to tumor eradication in almost all mice. These data are consistent with our recent evidence that pharmacological blockade of the COX-2/PGE_2_ axis enhances IFNγ production, effector T cell function and tumor control post-ICB^30^. Our present data indicates that for poorly immunogenic, treatment refractory tumors, dual combinations of CTX and ICB might be ineffective unless inhibitors of the COX-2/PGE_2_ axis are co-administered.

Our findings are of particular translational relevance given the current numerous clinical trials evaluating combinations of CTX and ICB and their approved use in various settings, including as first-line treatments in TNBC, urothelial and NSCLC patients^11,12,14,18^. Indeed, trials of certain combinations of CTX and ICB have recently failed to confirm benefit despite their accelerated approval status^70^, further highlighting the need for better combinations in the clinic. Our data are consistent with a model whereby dying cancer cell-derived PGE_2_ contributes to cytotoxic therapy failure by hindering the cancer-restraining T cell-mediated immune response that follows treatment. Lastly, the triple combination of CTX, ICB, and CXB uniquely impaired tumor regrowth and metastasis following resection of orthotopic breast tumors. This further supports the rationale for inhibiting the COX-2/PGE_2_ pathway alongside immunotherapy and cytotoxic therapy to improve the efficacy of this combination in both adjuvant and advanced disease settings.

## Methods

### Mice

All procedures involving animals were performed in accordance with the PDCC31AAF license approved by the Animal Welfare and Ethical Review Bodies (AWERB) of the CRUK Manchester Institute, and in accordance with National Home Office regulations under the Animals (Scientific Procedures) Act 1986. Female wild-type BALB/c mice aged 6-12 weeks (Envigo) and NSG mice aged 12 weeks (Charles River) were housed under specific pathogen-free conditions and in individually ventilated cages. Tumor volumes did not exceed 1500 mm^3^, the guideline set by the Committee of the National Cancer Research Institute^71^ as stipulated by the AWERB.

### Cell lines and cell culture

All cancer cell lines were cultured under standard conditions in RPMI-1640 (Lonza) supplemented with 1% Penicillin/Streptomycin (Thermo Fisher Scientific) and 10% fetal bovine serum (FBS; Life Technologies) and routinely confirmed to be mycoplasma-free (Venor® GeM gEP Mycoplasma Detection Kit, Minerva Biolabs) and mouse hepatitis virus-free (QIAamp® Viral RNA Mini extraction kit, Qiagen) by qPCR. CT26 COX-2^KO^ cells were generated by CRISPR/Cas9-mediated genome engineering as previously described^29^. 4T1 COX-2^KO^ cells were generated by ribonucleoprotein (RNP)-mediated CRISPR/Cas9-mediated editing (Integrated DNA Technologies) following manufacturer’s protocol. To produce the RNP complex, 1.5 pmol Cas9 enzyme was combined with 1.5 pmol crRNA:trRNA duplex (COX-2 crRNA: 5’-AGATGACTGCCCAACTCCCA-3’) in Opti-MEM media (Gibco) and incubated at room temperature for 5 min. The RNP complex was then combined with Lipofectamine RNAiMAX (Invitrogen) and Opti-MEM and incubated at room temperature for 20 min. 4T1 cells were trypsinized, washed with PBS and 4 × 10^4^ cells were added to the transfection mixture in 96-well plates. Cells were incubated for 48 h before being trypsinized and re-plated by single cell limiting dilution to obtain single cell clones. Lack of COX-2 expression and reduced PGE_2_ production was verified by western blotting using anti-COX-2 specific antibodies (Cell Signaling Technology) and by measuring the concentration of PGE_2_ in cell supernatants by ELISA (Cayman Chemical). To restore COX-2 expression in COX-2^KO^ cells, the full-length (1.8 kb) open reading frame of *Ptgs2* was cloned from the *Braf*^V600E^ 5555 melanoma cell line using the following primers (Fwd: 5’-TTTCCCGGATCCGCCACCATGCTCTTCCGAGCTGTGCT-3’; Rev: 5’-CCCTTTGTCGACTTACAGCTCAGTTGAACGCC-3’) and subcloned into the *BamHI* and *SalI* sites of the retroviral expression vector pFB-neo (Agilent). The resulting construct was confirmed by Sanger sequencing and co-transfected with the retroviral envelope vector pVSV-G into the viral packaging cell line GP2-293 (Takara Bio) with Lipofectamine 2000 (Invitrogen). 48 h post-transfection, the supernatant was used to transduce COX-2^KO^ cells and plates centrifuged at 1260 x g for 90 min at 32˚C to enhance transduction efficiency. Transduced cells were re-plated in 96-well plates in the presence of 300 μg/ml (4T1) or 600 μg/ml (CT26) G418 (Sigma) for 14 days to select for a single clone with COX-2 expression and PGE_2_ production levels comparable to parental cells. 4T1 COX-2^KO^ cells used in experiments alongside COX-2^REST^ cells had been transduced with an empty pFB-neo vector as a control.

### Subcutaneous 4T1 and CT26 tumor models

For inoculation into mice, 4T1 or CT26 cells were harvested in the exponential phase of growth by trypsinization (Sigma), washed three times with cold PBS (Thermo Fisher Scientific) and centrifugation steps at 300 x g for 5 min at 4˚C, filtered through a 70 µm cell strainer (Thermo Fisher Scientific) and resuspended in cold PBS. 5 × 10^5^ live cells were injected subcutaneously (s.c.) in 100 µl of PBS into the right flank of recipient mice. Tumor growth was monitored using a hand caliper and volume was calculated by measuring the longest diameter (length) and its perpendicular (width) using the formula: (length x width^2^)/2. On day 5-6 (4T1) or day 9-10 (CT26) post tumor cell injection, when tumors were approximately 100 mm^3^ or 75 mm^3^ respectively, mice were allocated to treatment arms by normalizing tumor size across groups.

### Orthotopic 4T1 tumor model

4T1 cells were harvested as above and 2 × 10^5^ live cells injected in 100 µl of PBS into the 4^th^ right inguinal mammary fatpad of recipient mice. On day 14 post-inoculation, tumors were surgically removed, at approximately 300 mm^3^. Mice were dosed s.c. with 0.05 ml/25 g of Buprecare (AnimalCare) 15 min prior to being anesthetized using isoflurane (4% in 100% oxygen at a flow rate of 4 L/min for induction and 2.5 L/min for maintenance). Mice were placed on a heat pad with integrated nasal airflow and their eyes protected from drying by Lacrilube (Allergan). The incision was injected with the local analgesic Marcaine and closed using coated Vicryl needle (Ethicon). Mice received 200 µl saline s.c. and were placed in a heated cage and monitored until they recovered from anesthesia. Two further doses of 0.05 ml/25 g Buprecare were administered 6-8 h post-surgery and the following day. Mice were fed mash diet following surgery and during CTX treatment. The day post-surgery, mice were allocated to treatment groups by normalizing tumor size prior to surgical resection (day 13 measurements) across groups. Mice were monitored for tumor regrowth at the orthotopic site and culled by a Schedule 1 method 2-4 weeks post-surgery and macroscopic lung nodules were counted by an investigator blinded to treatment group.

### In vivo treatments

Cisplatin (Sigma, P4394) was diluted in saline to a concentration of 1 mg/ml and 100 µl (5 mg/kg) administered intraperitoneally (i.p.) once weekly, for two doses. 5-FU (Sigma, F6627) was diluted in PBS to a concentration of 10 mg/ml and 100 µl (50 mg/kg) administered intraperitoneally (i.p.) once weekly, for two doses. Anti-PD-1 (clone RMP1-14, BioXCell) was diluted in PBS to a concentration of 2 mg/ml and 100 µl (200 µg per i.p. injection) administered twice weekly for 6 doses. Lyophilized celecoxib (CXB, LC Labs) was weighed using a fine balance and made up in a 60:40 ratio of DMSO (1 part, Sigma)/PEG400(5 parts, Sigma):dH_2_O at a concentration of 3 mg/ml. 200 µl (30 mg/kg) was given by oral gavage once daily or twice daily (see figure legends for details). Control treated mice received 100 µl saline or PBS once weekly for 2 doses, 100 µl PBS twice weekly for 6 doses and vehicle (60:40 ratio of DMSO:PEG:dH_2_O) by oral gavage once or twice daily.

### Peritoneal lavage: flow cytometry and analysis of soluble factors

4T1 tumor cells were treated with 50 µM cisplatin or 10 µM doxorubicin for 4 h or 100 µM 5-FU for 24 h, harvested by trypsinization (Sigma), washed 3 times with cold PBS (ThermoFisher) and centrifugation steps at 300 x g for 5 min at 4˚C, filtered through a 70 µm cell strainer (ThermoFisher) and resuspended in cold PBS. 5 × 10^6^ total cells were injected i.p. into mice in 200 µl PBS. Approximately 18 h later, animals were culled by Schedule 1 cervical dislocation and 5 ml PBS supplemented with EDTA (100 µM) was injected i.p. using a 27 G needle. The peritoneal cavity was massaged and a 25 G needle used to collect the lavage into tubes on ice. Recovered lavage volumes were recorded, cells pelleted by centrifugation (300 x g for 5 min at 4˚C) and resuspended in FACS buffer (PBS containing 1% FBS and 0.01% sodium azide) before filtering through a 70 µm cell strainer. Fc receptors were saturated with anti-CD16/32 (1:250, clone 93, eBioscience) 5 min before staining. Cell viability was determined by Aqua LIVE/Dead-405nm staining (Invitrogen). Samples were stained with combinations of the following antibodies: CD45-BV605 (Clone 30-F11, 1:200, #103140), CD11b-BV785 (Clone M1/70, 1:300, #101243), Siglec-F-BV711 (Clone E50-2440, 1:100, #740764), CD19-PE (Clone 1D3, 1:400, #557399), Ly6C-BV421 (Clone HK1.4, 1:400, #128032), Ly6G-FITC (Clone 1A8, 1:400, #127606), F4/80-PE-Cy7 (Clone BM8, 1:100, #123114), CD11c-AF700 (Clone N418, 1:200, #117320), MHCII I-A/I-E-PerCP-Cy5.5 (Clone M5/114.15.2, 1:300, #107626), CD49b-APC (Clone DX5, 1:100, #108910), CD3ε-PE-CF594 (Clone 145-2C11, 1:200, #100348) from eBioscience, BioLegend or BD Biosciences. Live cell counts were calculated from the acquisition of a fixed number (5000) of 10 μm latex beads (Beckman Coulter) mixed with a known volume of cell suspension and counts were normalized by the recovered lavage volume. Spectral overlap was calculated using live cells and cells were acquired on a Fortessa X-20 (BD Biosciences). For analysis of soluble factors, after removing cells by centrifugation, the peritoneal lavage was analyzed using mouse Simplex ProcartaPlex kits (Thermo Fisher Scientific) multiplexed to measure different cytokines, chemokines & growth factors. Samples were analyzed on a MAGPIX (Luminex).

### Flow cytometry of peripheral blood and tumor-infiltrating leukocytes

For analysis of peripheral blood leukocytes, 50 µl peripheral blood was taken from mice via tail-vein into an EDTA-coated capillary and then 1.5 ml tubes on ice. Samples were centrifuged at 300 x g for 6 min at 4˚C to separate plasma and cells, FACS buffer was added to the cell pellet and cell suspensions were moved to a 96-well V-bottom plate for antibody staining. Red blood cells were lysed using ACK buffer for 1 min (Gibco). For analysis of tumor-infiltrating leukocytes, tumors were collected into complete RPMI on ice. The surface of tumor samples were dried with paper and weights recorded. Samples were transferred into C-tubes (Miltenyi Biotech) containing RPMI and Collagenase IV (200 U/ml, Worthington Biochemical) and DNase I (0.2 mg/ml, Roche), then minced using scissors. The C-tubes were placed in a GentleMACS Octo Dissociator (Miltenyi Biotech), and tumors disaggregated with 2 rounds of the automated program m_impTumor_02_01. Dissociated tumors were incubated for 30 min at 37 °C and disaggregated for one more round. The C-tubes were centrifuged (300 x g for 5 min at 4˚C) and pellets resuspended in cold complete RPMI before being filtered through a 70 µm cell strainer and pelleted. Cell suspensions were resuspended in FACS buffer. Fc receptors were saturated with anti-CD16/32 (1:250, clone 93, eBioscience) 5 min before staining. Cell viability was determined by Aqua LIVE/Dead-405 nm staining (Invitrogen). Tumor or blood samples were stained with combinations of the following antibodies: CD45-BV605 (Clone 30-F11, 1:200, #103140), CD11b-BV785 (Clone M1/70, 1:300, #101243), Ly6G-PE-CF594 (Clone 1A8, 1:400, #127648), Ly6C-FITC (Clone AL-21, 1:400, #553104) or -BV421 (Clone HK1.4, 1:400, #128032), F4/80-PE-Cy7 (Clone BM8, 1:200, #123114), MHCII I-A/I-E APC-eFluor780 (Clone M5/114.15.2, 1:300, #47-5321-82), CD274(PD-L1)-PE (Clone MIH5, 1:100, #12-5982-82), CD19-PE (Clone 1D3, 1:400, #557399), CD49b-APC (Clone DX5, 1:100, #108910), CD3ε-PerCP-Cy5.5 (Clone 145-2C11, 1:100, #45-0031-82) or -PE-CF594 (Clone 145-2C11, 1:200, #100348), CD8α-PE (Clone 53-6.7, 1:100, #12-008182) or -PE-Cy7 (Clone 53-6.7, 1:200, #100722), CD4-FITC (Clone RM4-5, 1:300, #100510), CD44-APC-eFluor780 (Clone IM7, 1:100, #47-0441-82), IFNγ-eFluor450 (Clone XMG1.2, 1:80, #48-7311-82) from eBioscience, BioLegend or BD Biosciences. For intracellular cytokine detection, cells were stimulated ex vivo for 4 h with Cell Stimulation Cocktail (ThermoFisher) and stained using the Intracellular Fixation & Permeabilization Buffer Set (eBioscience) following manufacturer instructions. Monensin and Brefeldin A (both eBioscience) were added 2 h before the staining and non-specific binding of intracellular epitopes was blocked by pre-incubation of cells with 2% Normal Rat Serum (ThermoFisher). Live cell counts were calculated from the acquisition of a fixed number (5000) of 10 μm latex beads (Beckman Coulter) mixed with a known volume of cell suspension. Spectral overlap was calculated using live cells or VersaComp antibody capture beads (Beckman Coulter). Cells were acquired on a Novocyte (ACEA).

### Treatment of cancer cells in vitro

1 × 10^4^ (96-well plate for Incucyte experiments), 2 × 10^5^ (12-well plate for RNA) or 5 × 10^5^ cells (6-well plate for protein) were seeded overnight. The next day, the culture medium was replaced with fresh containing DMSO or CTX. All drug stocks were made up in DMSO, except for cisplatin which was reconstituted with saline and doxorubicin with dH_2_O. Cells were treated with 50 µM cisplatin (Sigma) or 100 µM 5-FU (Sigma), unless stated otherwise (see figure legends). For experiments using inhibitors, cells were treated alone or in combination with CTX using: 5 µM CXB (LC labs), 100 µM z-VAD-FMK (R&D systems) or 5 mM N-acetyl-L-cysteine (NAC, Sigma). For knockdown of transcription factors, 30 pmol of ON-TARGETplus siRNA from Horizon Discovery (Mouse Rela: #J-040776-05; J-040776-07; Cebpb: #J-043110-10; Jun: #J-043776-07; Sp1: #J-040633-21) or non-targeting siRNA (#D-001810-01-05) was mixed with 9 µl Lipofectamine RNAiMAX (Invitrogen) in 300 µl Opti-MEM (Gibco) and incubated at room temperature for 5 min. The transfection mixture was added to a 6-well plate and 5 × 10^5^ 4T1 cells added on top and gently mixed. Cells were incubated for 24 h, then the cell culture media was replaced with fresh media containing 100 µM 5-FU for 24 h. For irradiation of cells, 8 Gy of ionizing X-rays were administered using an Xstrahl CIX3 irradiator. For UV irradiation, the cell culture medium was changed to PBS prior to irradiation with 30mJ/cm^2^ UV-B using a Vilber Lourmat UV irradiation system. The PBS was replaced with RPMI-1640 medium immediately post-UV irradiation.

### ELISA

PGE_2_ levels in cell culture medium were measured following manufacturer’s instructions (Cayman Chemical). Samples were assayed neat or diluted to the linear range of known standards for detection.

### Western blotting

NP40 cell lysis buffer (Invitrogen) supplemented with PMSF (Sigma) and cOmplete protease inhibitor cocktail (Roche) was added directly to PBS-washed cells in a 6-well plate on ice, cells were scraped to collect lysates. Lysates were centrifuged at 21000 x g for 20 min at 4˚C and protein quantified using the Pierce BCA Protein Assay kit (Thermo Fisher Scientific). 25 µg of protein was diluted in laemmli buffer (Bio-Rad) with beta-mercaptoethanol, denatured at 95˚C for 5 min and loaded onto 10% Mini-PROTEAN TGX Gels (Bio-Rad). Proteins were transferred to nitrocellulose membranes using the Trans-Blot Turbo system (Bio-Rad) and membranes blocked with Intercept PBS blocking buffer (Li-COR) for 1 h at room temperature. Membranes were incubated overnight at 4˚C in Intercept PBS blocking buffer containing 0.2% Tween-20 with primary antibodies against COX-2 (D5H5, 1:1000, #12282), β-Tubulin (D3U1W, 1:2000, #86298), NF-κB p65 (D14E12, 1:2000, #8242), c-Jun (60A8, 1:1000, #9165), β-Actin (D6A8, 1:4000, #8457) all from Cell Signaling Technology, C/EBPβ (H-7, 1:500, #sc-7962) from Santa Cruz Biotechnology or Sp1 (1:10000, #NB600-232) from Novus Biologicals. Membranes were washed with PBS containing 0.1% Tween-20 and incubated with secondary antibodies IRDye 680RD Goat Anti-Mouse IgG and IRDye 800CW Goat Anti-Rabbit IgG (both 1:15000, Li-COR, #926-68070 and #926-32211) for 1 h at room temperature. Membranes were again washed with PBS containing 0.1% Tween-20 and protein bands were visualized using the Odyssey CLx system (Li-COR) and analyzed using Image Studio Lite (v5.2.5).

### Incucyte live-cell imaging

Imaging of cells was performed using an Incucyte S3 imaging system (Essen BioScience). Images were taken at 10X magnification, capturing 4 fields of view per well of a 96-well plate every 2 h. For kinetics of cell death, caspase-3/-7 green reagent (Essen BioScience) or Propidium Iodide (PI, Sigma) was added directly into the cell culture medium at final concentrations of 2.5 µM and 1 µg/ml respectively. For measuring ROS production, CellROX green reagent (Thermo Fisher Scientific) was added directly into the cell culture medium at a final concentration of 5 µM.

### Quantitative real-time PCR (qPCR)

Total RNA was extracted from cells using RLT lysis buffer (QIAGEN) and purified using RNeasy RNA isolation kit (QIAGEN). RNA was quantified using a NanoDrop One (ThermoFisher) and cDNA was synthesized by reverse transcription using High Capacity cDNA reverse transcription kit (Applied Biosystems) and quantitative real-time PCR was performed using TaqMan probes (Applied Biosystems) using a QS5 fast real-time PCR system (Applied Biosystems). Data were analyzed with the Δ2CT method. TaqMan probes used were: *Hprt* (*#*Mm03024075_m1*), Ptgs2* (#Mm00478374_m1), *Il6* (#Mm00446190_m1), *HPRT1* (#Hs02800695_m1) and *PTGS2* (#Hs00153133_m1).

### PrimeFlow RNA assay

4T1 cells were treated with 100 µM 5-FU for 24 h, trypsinized and collected into 96-well V-bottom plates for staining. Detection of *Ptgs2* mRNA was performed using PrimeFlow RNA assay kit (Thermo Fisher Scientific) with a type 1 probe set (Alexa Fluor 647) following manufacturer’s instructions. Cells were stained with Aqua LIVE/Dead-405 nm (Invitrogen) prior to fixing and probe hybridization. *Gapdh* mRNA was detected using a type 4 probe set (Alexa Fluor 488). Cells were acquired on a Fortessa X-20 (BD Biosciences).

### COX-2 GFP reporter cell line

4T1 COX-2 GFP reporter cells were generated by inserting the mCOX2 promoter fragment from pDRIVE5Lucia-mCOX2 (InvivoGen, #pdrive5lc-mcox2) and d2EGFP from pcDNA3.3-d2EGFP (Addgene, #26821) into the retroviral expression vector pFB-neo (Agilent) using Gibson assembly kit (New England Biolabs) and the following primers: pFB-neo_fwd tcct caatgtgtagTCCTCGAGCGGCCGC, pFB-neo_rev GCCCTGCAGGTCCGAATTCGTCGACA ATTCGATCCG, mCOX2 prom_fwd tgtcgacga CGAATTCGGACCTGCAGGGCCCACTAGT, mCOX2 prom_rev tgctcaccatGGCAGAGGTGGC, d2EGFP_fwd CACCTCTGCCatggtg agcaagggcg, and d2EGFP_rev CGCTCGAGGActacacattgatcctagcagaagcaca. The resulting pFB-neo-mCOX2p-d2EGFP construct was confirmed by Sanger sequencing and co-transfected with the retroviral envelope vector pVSV-G into the viral packaging cell line GP2-293 (Takara Bio) with Lipofectamine 2000 (Invitrogen). 48 h post-transfection, the supernatant was used to transduce 4T1 cells and plates centrifuged at 1260 x g for 90 min at 32˚C to enhance transduction efficiency. Transduced cells were re-plated in a 90 mm dish in the presence of 300 μg/ml G418 (Sigma) for 72 h.

### Compound library screen

4T1 COX-2 GFP reporter cells were seeded in black wall 384-well plates (Greiner) at a density of 2000 cells per well in 30 µl complete RPMI. Only the inner 240 wells were used per plate, with the outer two rows and columns filled with 30 µl PBS. Cells were left to adhere overnight and the next day the Prestwick Chemical Library containing 1280 small molecule drugs was dispensed using an Echo liquid handling machine (LabCyte). The 384-well plates with cells adhered were inverted over a source plate and compounds dispensed using acoustic energy to transfer 30 nl of compound into the cell culture medium, for a final concentration of 10 µM in 30 µl. DMSO (0.3%) was ran as a negative control and 100 µM 5-FU as a positive control, both dispensed in triplicate across each individual 384-plate in the upper left, middle and bottom right wells (total of nine DMSO or 5-FU control wells per plate) to account for potential intra-and inter-plate variation. Plates were imaged using an Incucyte S3 live-cell imaging system at 10X magnification with one image taken per well every 2 h over a 72 h period. A maximum of 6 plates were run at one-time, therefore to cover the total compound library three separate runs were performed over the course of two weeks. Antineoplastic agents were re-run in a subsequent screen to confirm the obtained results. To analyse the data, analysis settings were defined as described below and applied across all images. The mask for analysis of cell confluence had an area filter of >250 µm^2^ to exclude cell debris. GFP fluorescence analysis mask used Top-Hat background subtraction with radius 10 µm and threshold of 0.2 Green Calibrated Units (GCU) and area filter >80 µm^2^ to detect GFP + objects (1 cell or a small cluster of neighboring cells). Using the Incucyte analysis software, the average pixel intensity in GCU within a GFP + object was calculated, then the mean of all GFP + objects across the image/well used to generate the mean intensity value per well. Raw mean intensity and percent confluency data were exported and used for downstream analysis. To calculate GFP and confluency scores per compound, the mean of all nine DMSO control wells situated across the plate was calculated per time-point and the fold change in confluency or GFP over time was computed per compound. For GFP scores, the area under the curve (AUC) of the fold change in mean intensity over time was calculated. Confluency scores were generated by calculating the mean of the fold change in confluency from DMSO controls over the whole culture time. Eight compounds were excluded from the analysis due to autofluorescence: Merbromin, Verteporfin, Pyrvinium pamoate, 1,8-Dihydroxyanthraquinone, Dipyridamole, Quinacrine dihydrochloride hydrate, Propidium iodide and Chicago sky blue 6B.

### Analysis of the NCI-60 human cancer cell line dataset

Transcriptomic data collected from untreated control and CTX-treated cells at 2 h, 6 h and 24 h were downloaded from NCBI Gene Expression Omnibus database through the GEO reference series GSE116436 related to the NCI Transcriptional Pharmacodynamics Workbench (NCI TPW)^34^. In accordance with the NCI TPW, gene expression fold changes are defined as the relative difference in log2 expression between treated and corresponding untreated cells at each time-point. Log10 GI_50_ data defining drug sensitivity were also obtained from the NCI TPW portal (https://tpwb.nci.nih.gov). The highest drug concentration was used for analysis: gemcitabine (2 µM), cisplatin (15 µM), topotecan (1 µM), doxorubicin (1 µM) and paclitaxel (0.1 µM).

### Statistical analysis

Data were analyzed using Microsoft Excel 2019 and graphs were plotted using GraphPad Prism v9.1.2 (GraphPad Software Inc.). Flow cytometry standard (.fcs) files were analyzed using FlowJo v10.8.0 (Tree Star Inc.). Incucyte images were analyzed using Incucyte software GUI v2020C Rev1 (Essen BioScience). Statistics were calculated with GraphPad Prism and values expressed as mean ± SEM. Data were analyzed with the following tests (see figure legends for details): Unpaired two-tailed Student’s *t*-test, Log-rank (Mantel-Cox) test, one-way ANOVA tests adjusted for multiple comparisons using Tukey’s test, Kruskal-Wallis test with Dunn’s multiple comparisons test for non-Gaussian distributed data, two-way ANOVA or mixed-effects model analysis with Sidak’s multiple comparisons. A *p* value < 0.05 (*p < 0.05, **p < 0.01, ***p < 0.001, ****p < 0.0001) was considered significant. Exact *p* values are provided in the Source Data file.

### Reporting summary

Further information on research design is available in the Nature Research Reporting Summary linked to this article.

## Supplementary information

Supplementary information

Reporting Summary

## Data Availability

The NCI-60 human cancer cell publically available data used in this study are available in the NCBI Gene Expression Omnibus database under accession code GSE116436. Source data are provided with this paper. The relevant data supporting the findings in this study are available in the Article, Supplementary Information, or Source Data file. Source data are provided with this paper.

## Source data

Source data file

## References

[1] Ribas A, Wolchok JD (2018). Cancer immunotherapy using checkpoint blockade. Science.

[2] Rodriguez-Ruiz ME, Vitale I, Harrington KJ, Melero I, Galluzzi L (2020). Immunological impact of cell death signaling driven by radiation on the tumor microenvironment. Nat. Immunol..

[3] Galluzzi L, Humeau J, Buqué A, Zitvogel L, Kroemer G (2020). Immunostimulation with chemotherapy in the era of immune checkpoint inhibitors. Nat. Rev. Clin. Oncol..

[4] Galluzzi L, Buqué A, Kepp O, Zitvogel L, Kroemer G (2017). Immunogenic cell death in cancer and infectious disease. Nat. Rev. Immunol..

[5] Giampazolias E (2017). Mitochondrial permeabilization engages NF-κB-dependent anti-tumour activity under caspase deficiency. Nat. Cell Biol..

[6] Yatim N (2015). RIPK1 and NF-κB signaling in dying cells determines cross-priming of CD8+ T cells. Science.

[7] Zelenay S (2013). & Reis e Sousa, C. Adaptive immunity after cell death. Trends Immunol..

[8] Cubas R (2018). Chemotherapy combines effectively with anti-PD-L1 treatment and can augment antitumor responses. J. Immunol..

[9] Ariyan CE (2018). Robust antitumor responses result from local chemotherapy and CTLA-4 blockade. Cancer Immunol. Res..

[10] Pfirschke C (2016). Immunogenic chemotherapy sensitizes tumors to checkpoint blockade therapy. Immunity.

[11] Salas-Benito D (2021). Paradigms on immunotherapy combinations with chemotherapy. Cancer Discov..

[12] Gandhi L (2018). Pembrolizumab plus chemotherapy in metastatic non–small-cell lung cancer. N. Engl. J. Med..

[13] West H (2019). Atezolizumab in combination with carboplatin plus nab-paclitaxel chemotherapy compared with chemotherapy alone as first-line treatment for metastatic non-squamous non-small-cell lung cancer (IMpower130): a multicentre, randomised, open-label, phase 3 trial. Lancet Oncol..

[14] Galsky MD (2020). Atezolizumab with or without chemotherapy in metastatic urothelial cancer (IMvigor130): a multicentre, randomised, placebo-controlled phase 3 trial. Lancet.

[15] Savas P, Loi S (2020). Expanding the role for immunotherapy in triple-negative breast cancer. Cancer Cell.

[16] Schmid P (2020). Pembrolizumab for early triple-negative breast cancer. N. Engl. J. Med..

[17] Schmid P (2020). Atezolizumab plus nab-paclitaxel as first-line treatment for unresectable, locally advanced or metastatic triple-negative breast cancer (IMpassion130): updated efficacy results from a randomised, double-blind, placebo-controlled, phase 3 trial. Lancet Oncol..

[18] Narayan P (2020). FDA approval summary: atezolizumab plus paclitaxel protein-bound for the treatment of patients with advanced or metastatic TNBC whose tumors express PD-L1. Clin. Cancer Res..

[19] Chan KS (2016). Molecular pathways: targeting cancer stem cells awakened by chemotherapy to abrogate tumor repopulation. Clin. Cancer Res..

[20] Jiang M-J, Gu D-N, Dai J-J, Huang Q, Tian L (2020). Dark side of cytotoxic therapy: chemoradiation-Induced cell death and tumor repopulation. Trends Cancer.

[21] Révész L (1956). Effect of tumour cells killed by X-rays upon the growth of admixed viable cells. Nature.

[22] Sulciner ML (2018). Resolvins suppress tumor growth and enhance cancer therapy. J. Exp. Med..

[23] Hou J, Greten TF, Xia Q (2017). Immunosuppressive cell death in cancer. Nat. Rev. Immunol..

[24] Ichim G, Tait SWG (2016). A fate worse than death: apoptosis as an oncogenic process. Nat. Rev. Cancer.

[25] Madden EC, Gorman AM, Logue SE, Samali A (2020). Tumour cell secretome in chemoresistance and tumour recurrence. Trends Cancer.

[26] Kim JJ, Tannock IF (2005). Repopulation of cancer cells during therapy: an important cause of treatment failure. Nat. Rev. Cancer.

[27] Bonavita E (2020). Antagonistic inflammatory phenotypes dictate tumor fate and response to immune checkpoint blockade. Immunity.

[28] Böttcher JP (2018). NK cells stimulate recruitment of cDC1 into the tumor microenvironment promoting cancer immune control. Cell.

[29] Zelenay S (2015). Cyclooxygenase-dependent tumor growth through evasion of immunity. Cell.

[30] Pelly VS (2021). Anti-inflammatory drugs remodel the tumor immune environment to enhance immune checkpoint blockade efficacy. Cancer Discov..

[31] Hangai S (2016). PGE2 induced in and released by dying cells functions as an inhibitory DAMP. Proc. Natl Acad. Sci. USA.

[32] Huang Q (2011). Caspase 3-mediated stimulation of tumor cell repopulation during cancer radiotherapy. Nat. Med..

[33] Kurtova AV (2015). Blocking PGE2-induced tumour repopulation abrogates bladder cancer chemoresistance. Nature.

[34] Monks A (2018). The NCI Transcriptional Pharmacodynamics Workbench: a tool to examine dynamic expression profiling of therapeutic response in the NCI-60 cell line panel. Cancer Res..

[35] Yang H (2018). The role of cellular reactive oxygen species in cancer chemotherapy. J. Exp. Clin. Cancer Res..

[36] Onodera Y, Teramura T, Takehara T, Shigi K, Fukuda K (2015). Reactive oxygen species induce Cox-2 expression via TAK1 activation in synovial fibroblast cells. FEBS Open Bio..

[37] Kang YJ, Mbonye UR, DeLong CJ, Wada M, Smith WL (2007). Regulation of intracellular cyclooxygenase levels by gene transcription and protein degradation. Prog. Lipid Res..

[38] Subbaramaiah K, Dannenberg AJ (2003). Cyclooxygenase 2: a molecular target for cancer prevention and treatment. Trends Pharmacol. Sci..

[39] Kirkby NS (2016). Systematic study of constitutive cyclooxygenase-2 expression: Role of NF-κB and NFAT transcriptional pathways. Proc. Natl Acad. Sci. USA.

[40] Feng X (2017). Dying glioma cells establish a proangiogenic microenvironment through a caspase 3 dependent mechanism. Cancer Lett..

[41] Yamamoto M, Alemany R, Adachi Y, Grizzle WE, Curiel DT (2001). Characterization of the cyclooxygenase-2 promoter in an adenoviral vector and its application for the mitigation of toxicity in suicide gene therapy of gastrointestinal cancers. Mol. Ther..

[42] Dogra N, Kumar A, Mukhopadhyay T (2018). Fenbendazole acts as a moderate microtubule destabilizing agent and causes cancer cell death by modulating multiple cellular pathways. Sci. Rep..

[43] Varbanov HP, Kuttler F, Banfi D, Turcatti G, Dyson PJ (2017). Repositioning approved drugs for the treatment of problematic cancers using a screening approach. PLoS ONE.

[44] Chen C-J (2007). Identification of a key pathway required for the sterile inflammatory response triggered by dying cells. Nat. Med..

[45] Eigenbrod T, Park J-H, Harder J, Iwakura Y, Núñez G (2008). Cutting edge: critical role for mesothelial cells in necrosis-induced inflammation through the recognition of IL-1 alpha released from dying cells. J. Immunol..

[46] Teijeira Á (2020). CXCR1 and CXCR2 chemokine receptor agonists produced by tumors induce neutrophil extracellular traps that interfere with immune cytotoxicity. Immunity.

[47] De Henau O (2016). Overcoming resistance to checkpoint blockade therapy by targeting PI3Kγ in myeloid cells. Nature.

[48] Hayashi K (2020). Tipping the immunostimulatory and inhibitory DAMP balance to harness immunogenic cell death. Nat. Commun..

[49] Altorki NK (2005). Chemotherapy induces the expression of cyclooxygenase-2 in non-small cell lung cancer. Clin. Cancer Res..

[50] Veglia F (2019). Fatty acid transport protein 2 reprograms neutrophils in cancer. Nature.

[51] Roulis M (2020). Paracrine orchestration of intestinal tumorigenesis by a mesenchymal niche. Nature.

[52] Kalinski P (2012). Regulation of immune responses by prostaglandin E2. J. Immunol..

[53] Wang D, Cabalag CS, Clemons NJ, DuBois RN (2021). Cyclooxygenases and prostaglandins in tumor immunology and microenvironment of gastrointestinal cancer. Gastroenterology.

[54] Gonçalves S (2021). COX2 regulates senescence secretome composition and senescence surveillance through PGE2. Cell Rep..

[55] Jaillon S (2020). Neutrophil diversity and plasticity in tumour progression and therapy. Nat. Rev. Cancer.

[56] Wculek SK, Malanchi I (2015). Neutrophils support lung colonization of metastasis-initiating breast cancer cells. Nature.

[57] Coffelt SB (2015). IL-17-producing γδ T cells and neutrophils conspire to promote breast cancer metastasis. Nature.

[58] Szczerba BM (2019). Neutrophils escort circulating tumour cells to enable cell cycle progression. Nature.

[59] Spiegel A (2016). Neutrophils suppress intraluminal NK cell-mediated tumor cell clearance and enhance extravasation of disseminated carcinoma cells. Cancer Discov..

[60] Kowanetz M (2010). Granulocyte-colony stimulating factor promotes lung metastasis through mobilization of Ly6G+Ly6C+ granulocytes. Proc. Natl Acad. Sci. USA.

[61] Acharyya S (2012). A CXCL1 paracrine network Links cancer chemoresistance and metastasis. Cell.

[62] Xiao Y (2021). Cathepsin C promotes breast cancer lung metastasis by modulating neutrophil infiltration and neutrophil extracellular trap formation. Cancer Cell.

[63] Albrengues J (2018). Neutrophil extracellular traps produced during inflammation awaken dormant cancer cells in mice. Science.

[64] Finisguerra V (2015). MET is required for the recruitment of anti-tumoural neutrophils. Nature.

[65] Chang Cui A (2021). Neutrophil elastase selectively kills cancer cells and attenuates tumorigenesis. Cell.

[66] Fridlender ZG (2009). Polarization of tumor-associated neutrophil phenotype by TGF-β: ‘N1’ versus ‘N2’ TAN. Cancer Cell.

[67] Hagerling C (2019). Immune effector monocyte–neutrophil cooperation induced by the primary tumor prevents metastatic progression of breast cancer. Proc. Natl Acad. Sci. USA.

[68] Li P (2020). Dual roles of neutrophils in metastatic colonization are governed by the host NK cell status. Nat. Commun..

[69] Galdiero MR (2016). Occurrence and significance of tumor-associated neutrophils in patients with colorectal cancer. Int. J. Cancer.

[70] Beaver JA, Pazdur R (2021). “Dangling” accelerated approvals in oncology. N. Engl. J. Med..

[71] Workman P (2010). Guidelines for the welfare and use of animals in cancer research. Br. J. Cancer.

