# Supplementary material for: Chemotherapy-induced COX-2 upregulation by cancer cells defines their inflammatory properties and limits the efficacy of chemoimmunotherapy combinations
Source: Nat Commun. 2022 Apr 19;13:2063. doi: 10.1038/s41467-022-29606-9 (PMC9018752; doi:10.1038/s41467-022-29606-9)
Supplement: Supplementary file 1 — Supplementary information [file 41467_2022_29606_MOESM1_ESM.pdf]

Supplementary Figure 1

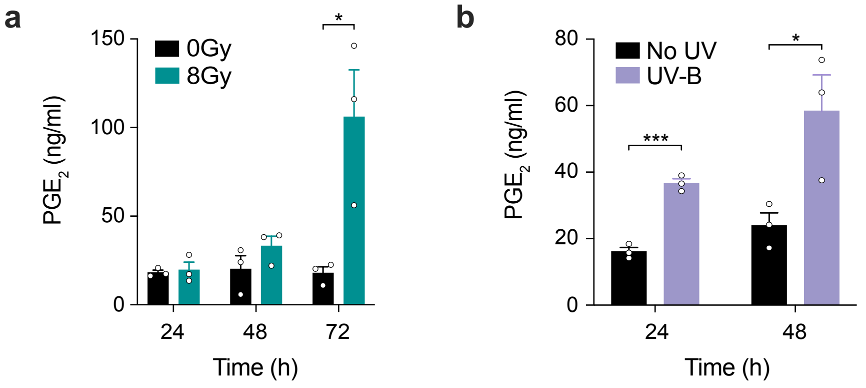

Supplementary Figure 1 (related to Figure 1). Irradiation of tumor cells enhances PGE_2 release. 4T1 cells were irradiated with 8 Gy ionizing X-rays (**a**) or 30 mJ/cm^2 UV-B (**b**) and PGE_2 release into the cell culture medium was measured over time. Data are represented as mean ±SEM of $n = 3$ independent experiments, *$p < 0.05$, ***$p < 0.001$ as determined by unpaired two-tailed t-test. Source data and exact p values are provided as a Source Data file.

Supplementary Figure 2

a

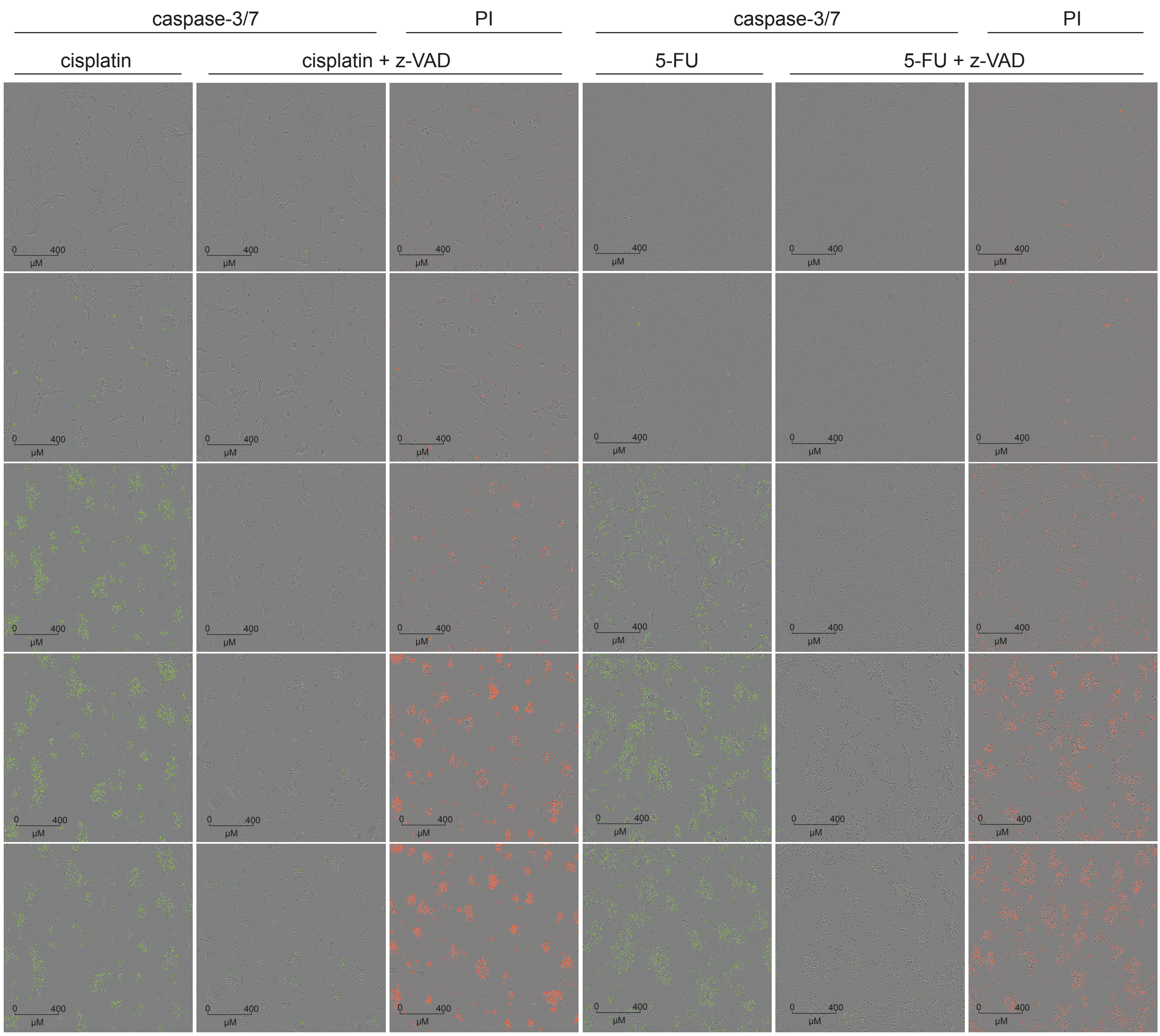

b

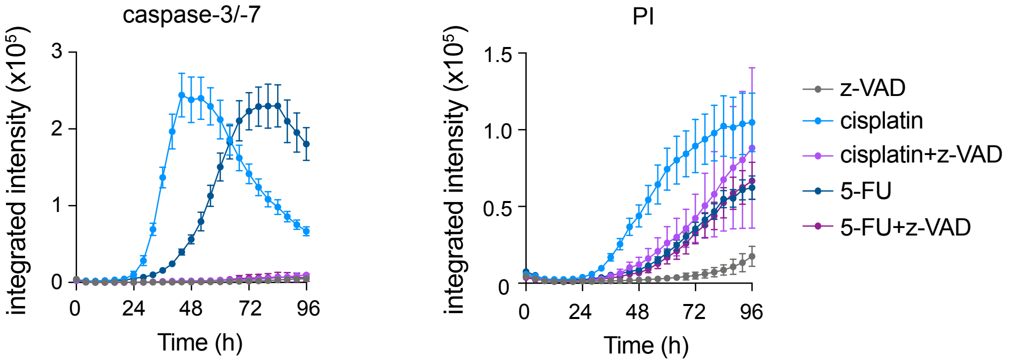

Supplementary Figure 2 (related to Figure 2). Cell death kinetics and caspase-3/-7 activation in chemotherapy treated cells. (**a**) Representative images of 4T1 tumor cells treated with cisplatin (50 µM) or 5-FU (100 µM) in the presence or absence of pan-caspase inhibitor z-VAD-FMK (z-VAD, 100 µM). Images are representative of triplicate wells with four fields of view, cells were stained with caspase-3/-7 green reagent or propidium iodide (PI). (**b**) Quantification of caspase-3/-7 and PI, data represented as mean ±SD of triplicate wells, representative of *n* = 2 independent experiments. Source data are provided as a Source Data file.

Supplementary Figure 3

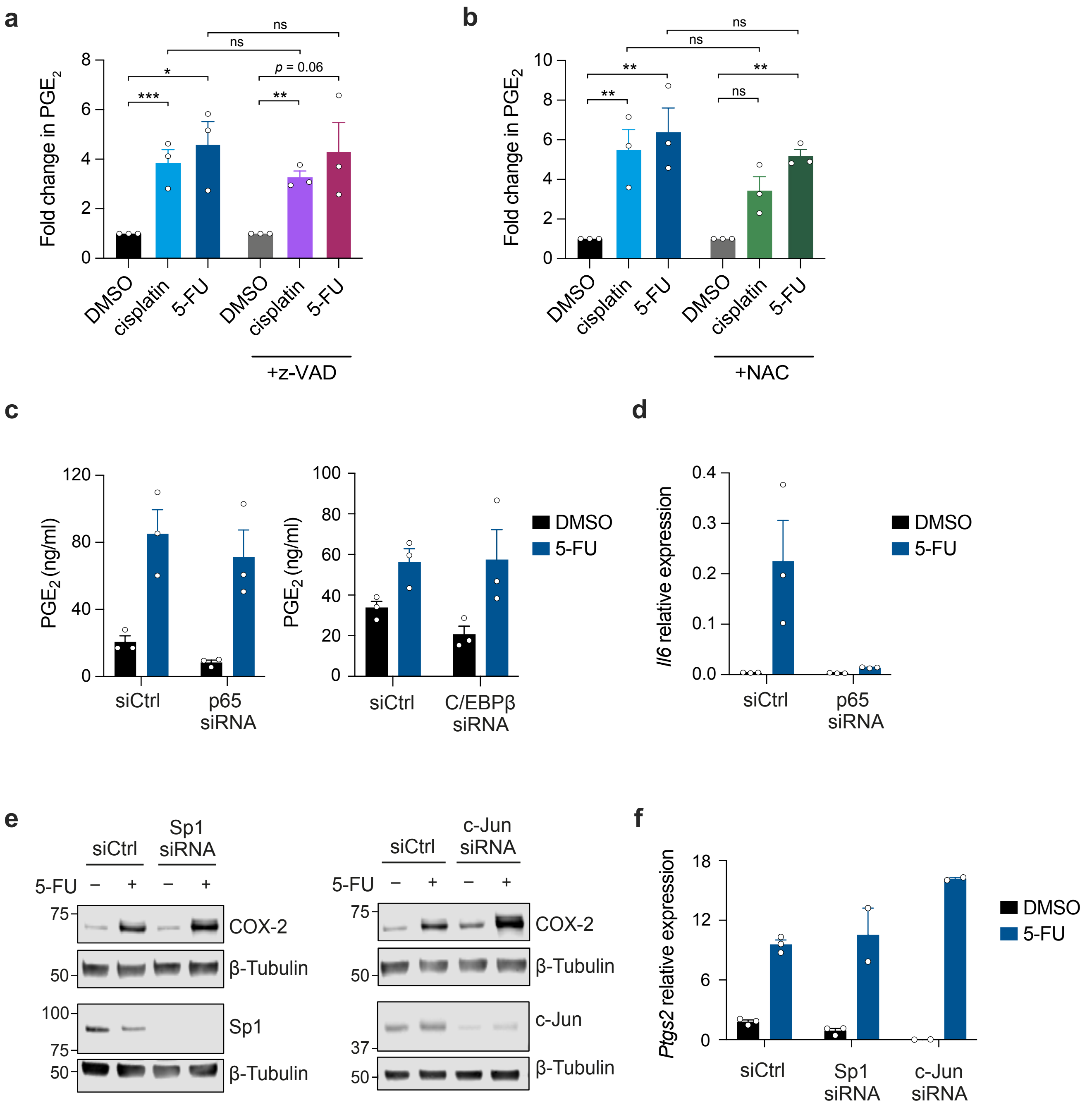

Supplementary Figure 3 (related to Figure 2). Chemotherapy-induced COX-2/PGE_2 does not solely depend on caspase activity, ROS production, or the transcription factors NF-κB, C/EBPβ, Sp1 or c-Jun. (**a-b**) Fold change in PGE_2 release from 4T1 tumor cells treated with cisplatin (50 μM) for 8h or 5-FU (100 μM) for 24h in the presence or absence of pan-caspase inhibitor z-VAD-FMK (z-VAD, 100 μM) (**a**) or ROS scavenger N-acetyl-L-cysteine (NAC, 5 mM) (**b**). Data are represented as mean ±SEM of $n = 3$ independent experiments. *$p < 0.05$, **$p < 0.01$, ***$p < 0.001$ as determined by one-way ANOVA with Tukey's multiple comparisons test. (**c**) PGE_2 release from 4T1 cells treated with DMSO or 5-FU (100 μM) for 24h. Cells were transfected with control or siRNA targeting p65 or C/EBPβ 24h prior to 5-FU treatment. Mean ±SEM of triplicate wells. (**d**) *Il6* expression relative to *Hprt* in 4T1 cells treated with DMSO or 5-FU (100 μM) for 24h. Mean ±SEM of triplicate wells. (**e-f**) COX-2 protein (**e**) and mRNA (relative to *Hprt*) levels (**f**) in 4T1 cells following 24h 5-FU (100 μM) treatment. Cells were transfected with control or siRNA targeting Sp1 or c-Jun 24h prior to 5-FU treatment. Mean ±SEM and westerns representative of duplicate or triplicate wells. β-Tubulin loading controls from the same membrane are shown. Source data and exact *p* values are provided as a Source Data file.

Supplementary Figure 4

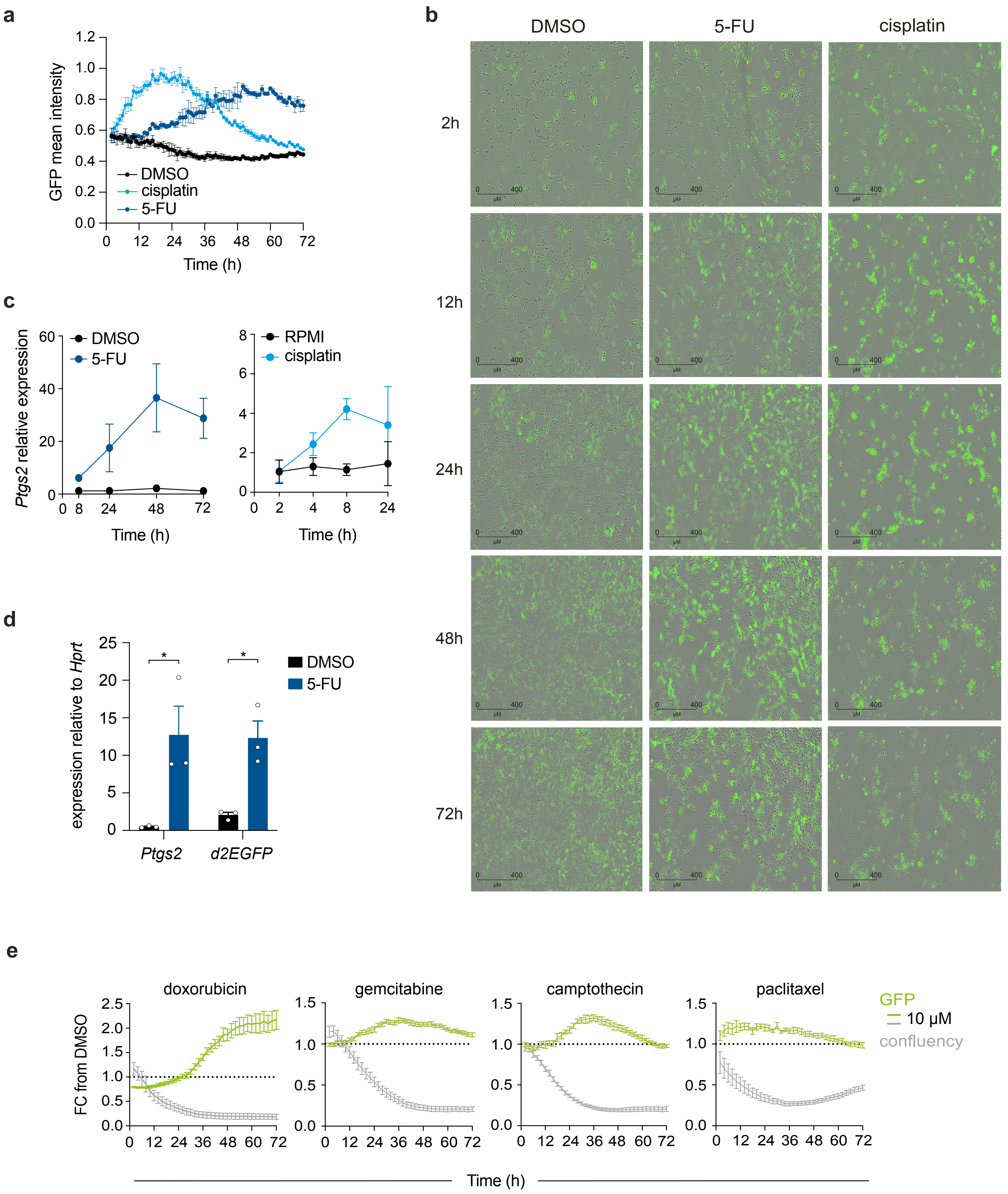

Supplementary Figure 4 (related to Figure 3). Characterization of the 4T1 COX-2 GFP reporter cell line. (**a**) Mean intensity of GFP over time in cells treated with DMSO, cisplatin (50 μM) or 5-FU (100 μM). Data are represented as mean ±SD of triplicate wells. (**b**) Representative images of 4T1 COX-2 GFP reporter cells treated with DMSO, 5-FU (100 μM) or cisplatin (50 μM) over time. Images are representative of triplicate wells with one field of view. (**c**) Change in *Ptgs2* expression relative to *Hprt* over time in 4T1 cells treated with DMSO, 5-FU (100 μM) or cisplatin (50 μM). Data are represented as mean ±SEM of $n = 2$ independent experiments. (**d**) Expression of endogenous *Ptgs2* transcripts and destabilized GFP (*d2EGFP*) transcripts in 4T1 COX-2 GFP reporter cells treated with 5-FU (100 μM) for 48h. Data are represented as mean ±SEM of $n = 3$ independent experiments, $^*p < 0.05$ as determined by unpaired two-tailed t-test. (**e**) Fold change in GFP mean intensity and confluency from DMSO-treated controls over time, mean ±SEM of six wells from $n = 2$ independent experiments. Source data and exact *p* values are provided as a Source Data file.

Supplementary Figure 5

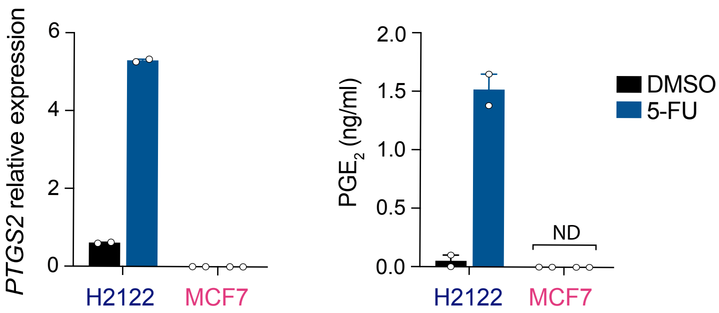

Supplementary Figure 5 (related to Figure 4). 5-FU upregulates COX-2 and increases PGE_2 release from human tumor cells with baseline *PTGS2* expression. NCI-H2122 (H2122) lung carcinoma cells and MCF7 breast carcinoma cells were treated with 5-FU (100 μM) for 24h and *PTGS2* expression relative to *HPRT1* and PGE_2 release into the cell culture medium were measured. Blue indicates cell line is *PTGS2* positive at baseline and pink indicates *PTGS2* negative. ND = not detected. Mean ±SEM of duplicate wells, representative plot shown of $n = 2$ independent experiments. Source data are provided as a Source Data file.

Supplementary Figure 6

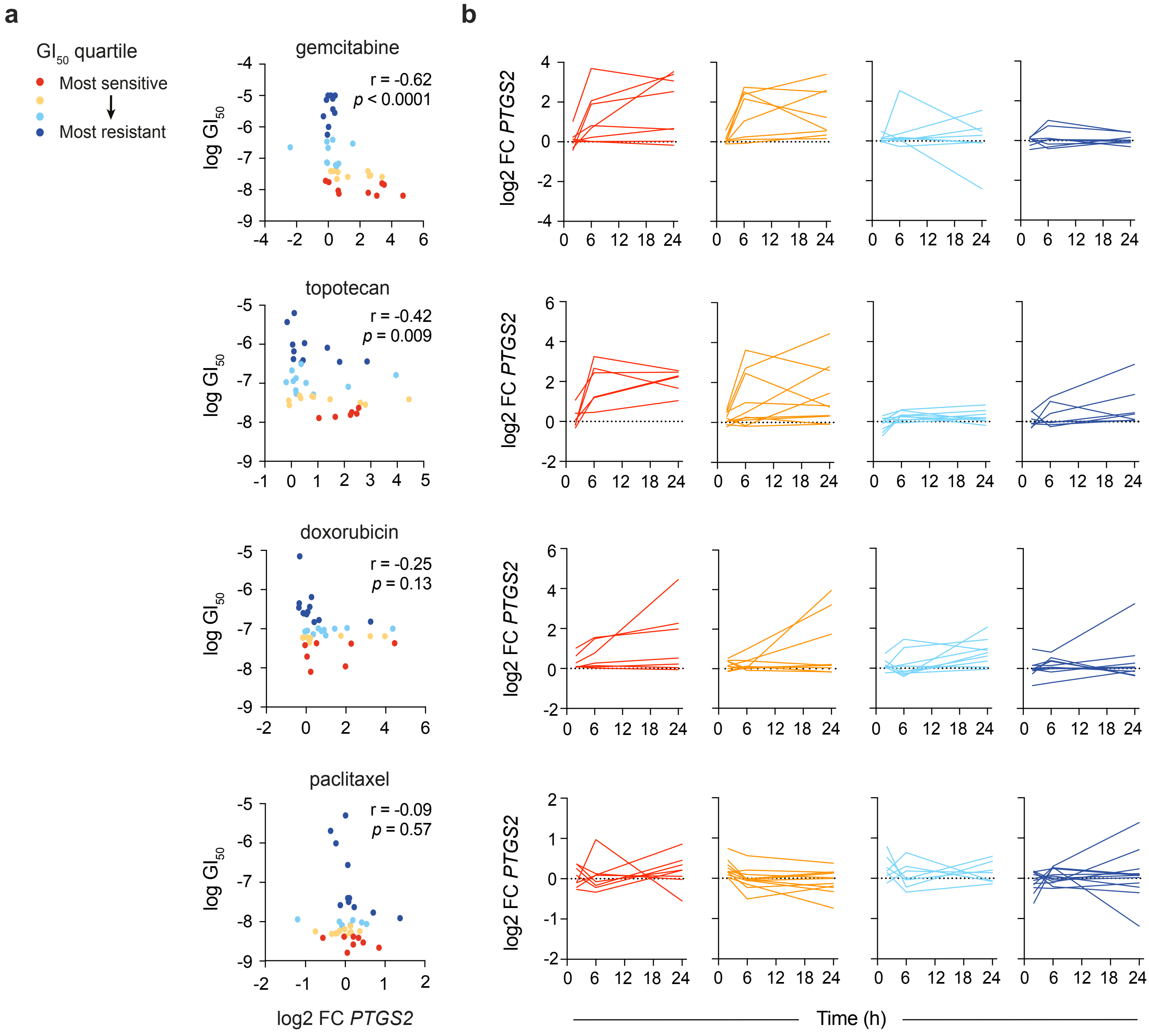

Supplementary Figure 6 (related to Figure 4). Relationship between drug sensitivity and upregulation of *PTGS2* in the human NCI-60 cancer cell line panel. (**a**) Dot plots showing log2 fold change in *PTGS2* at 24h against log10 GI_{50} values for different chemotherapy drugs. Cell lines with available GI_{50} data (*n* = 40 for all) were separated into quartiles with the most sensitive (red) to most resistant (dark blue) shown. Spearman's rank correlation coefficient and *p* value is shown. (**b**) Log2 fold change in *PTGS2* values over time for cell lines grouped based on GI_{50} as in **a**. Most sensitive Q1 (red, gemcitabine and paclitaxel *n* = 8, topotecan *n* = 6, doxorubicin *n* = 7), Q2 (orange, gemcitabine *n* = 8, topotecan *n* = 10, doxorubicin *n* = 9, paclitaxel *n* = 11), Q3 (light blue, gemcitabine *n* = 8, topotecan and doxorubicin *n* = 9, paclitaxel *n* = 7), most resistant Q4 (dark blue, gemcitabine *n* = 8, topotecan *n* = 7, doxorubicin *n* = 10, paclitaxel *n* = 12). Source data are provided as a Source Data file.

Supplementary Figure 7

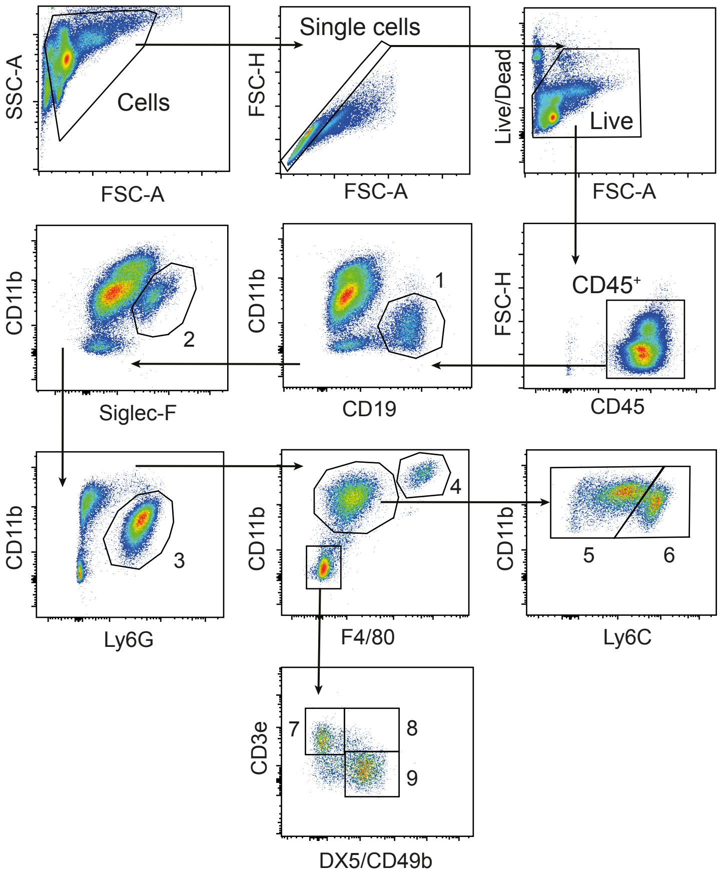

1 - CD11b$^-$ CD19$^+$ (B cells)
2 - CD11b^{int} Siglec-F$^+$ (Eosinophils)
3 - CD11b$^+$ Ly6G$^+$ (Neutrophils)
4 - CD11b$^+$ F4/80^{hi} (LPM)
5 - CD11b$^+$ F4/80^{int} Ly6C$^{int/lo}$ (SPM)
6 - CD11b$^+$ F4/80^{int} Ly6C^{hi} (Monocytes)
7 - CD3e$^+$ (T cells)
8 - CD3e$^+$ DX5/CD49b$^+$ (NKT cells)
9 - CD3e$^-$ DX5/CD49b$^+$ (NK cells)

Supplementary Figure 7 (related to Figure 5). Flow cytometry gating strategy for peritoneal lavage analysis. Representative gating strategy and cell definitions by surface markers for data shown in Figure 5. LPM = large peritoneal macrophages, SPM = small peritoneal macrophages.

Supplementary Figure 8

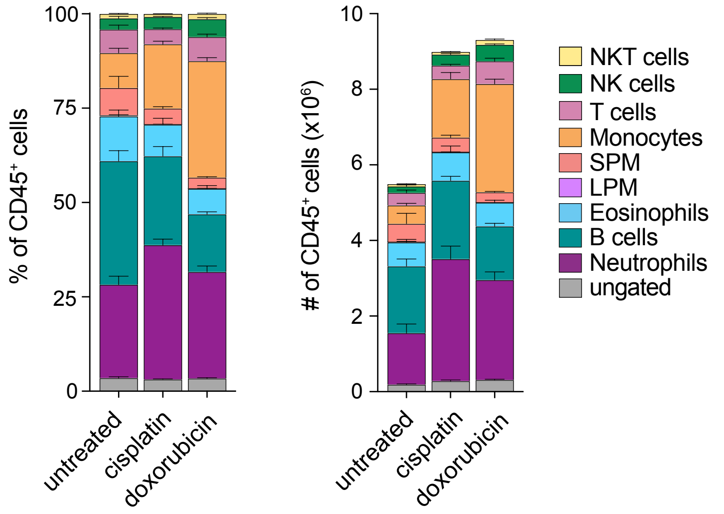

Supplementary Figure 8 (related to Figure 5). Chemotherapy pre-treatment of tumor cells increases immune cell recruitment in a peritoneal injection model. Frequency (left panel) and total number (right panel) of live $CD45^+$ immune cells present in mice injected with untreated, cisplatin or doxorubicin pre-treated 4T1 cells. Data are represented as mean ±SEM, *n* = 4 (cisplatin) or 5 (control, doxorubicin) mice per group. LPM = large peritoneal macrophages, SPM = small peritoneal macrophages. Source data are provided as a Source Data file.

Supplementary Figure 9

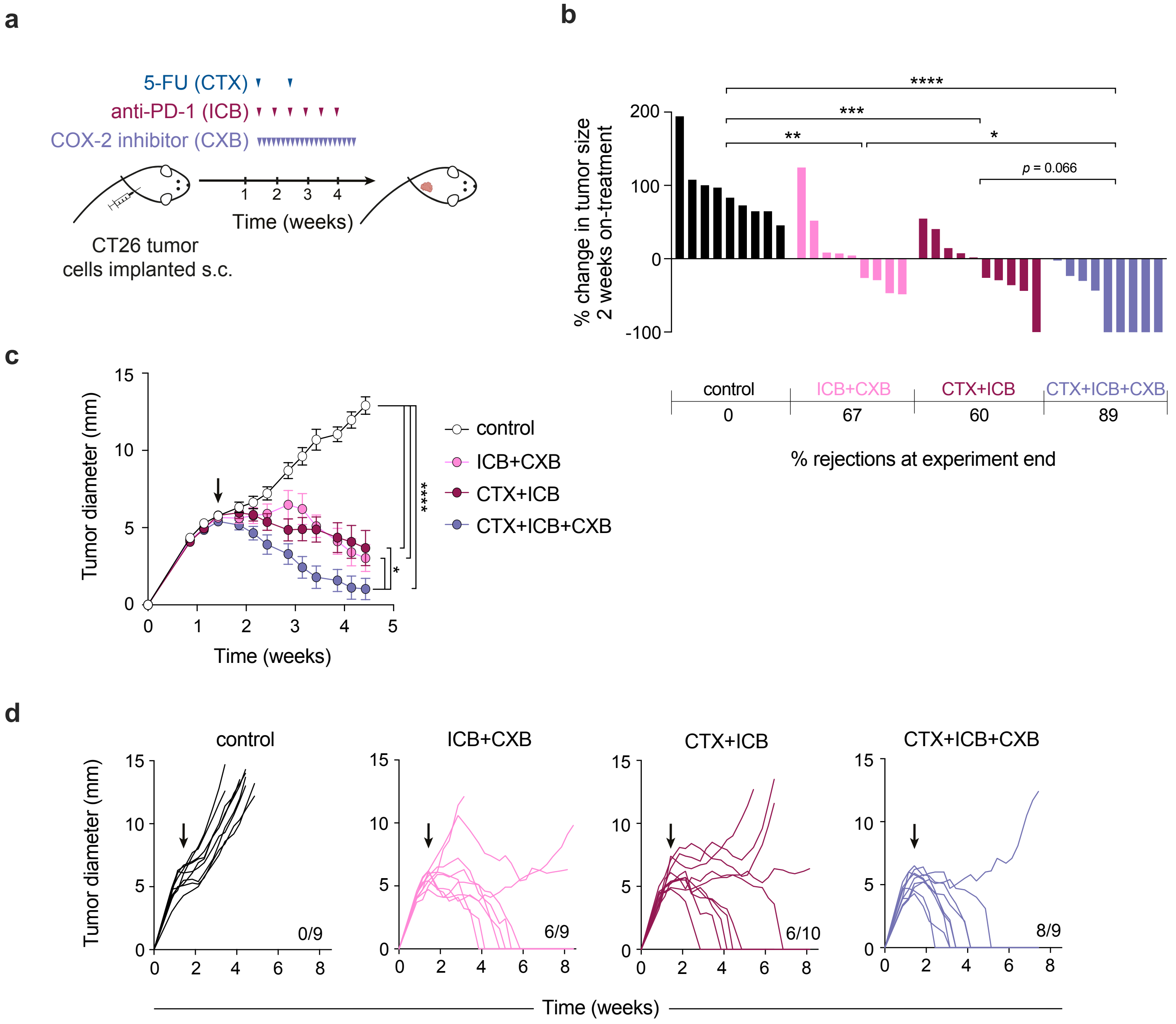

Supplementary Figure 9 (related to Figure 6). COX-2 inhibition enhances tumor control during chemotherapy and immunotherapy combination treatment. (**a**) Mice were inoculated subcutaneously with CT26 tumor cells and treatment began on day 9-10 post-injection when tumor volumes were 72.0 ±4.9mm^3 (mean ±SEM). (**b**) Waterfall plot showing percent change in tumor size two weeks post-treatment start, each bar represents one mouse (*n* = 9 (control, ICB+CXB, CTX+ICB+CXB) or 10 (CTX+ICB) mice per group, pool of two independent experiments). Percent of tumor rejections at the experiment end is shown. (**c-d**) Mean (**c**) or individual (**d**) tumor growth profiles for mice shown in **b**. Arrow indicates treatment start, mice received CXB or vehicle treatment bidaily. Number of tumor rejections at the experiment end out of total animals treated is shown. *$p < 0.05$, **$p < 0.01$, ***$p < 0.001$, ****$p < 0.0001$ as determined by one-way ANOVA with Tukey's multiple comparisons test (**b**) or two-way ANOVA (**c**). Source data and exact *p* values are provided as a Source Data file.

Supplementary Figure 10

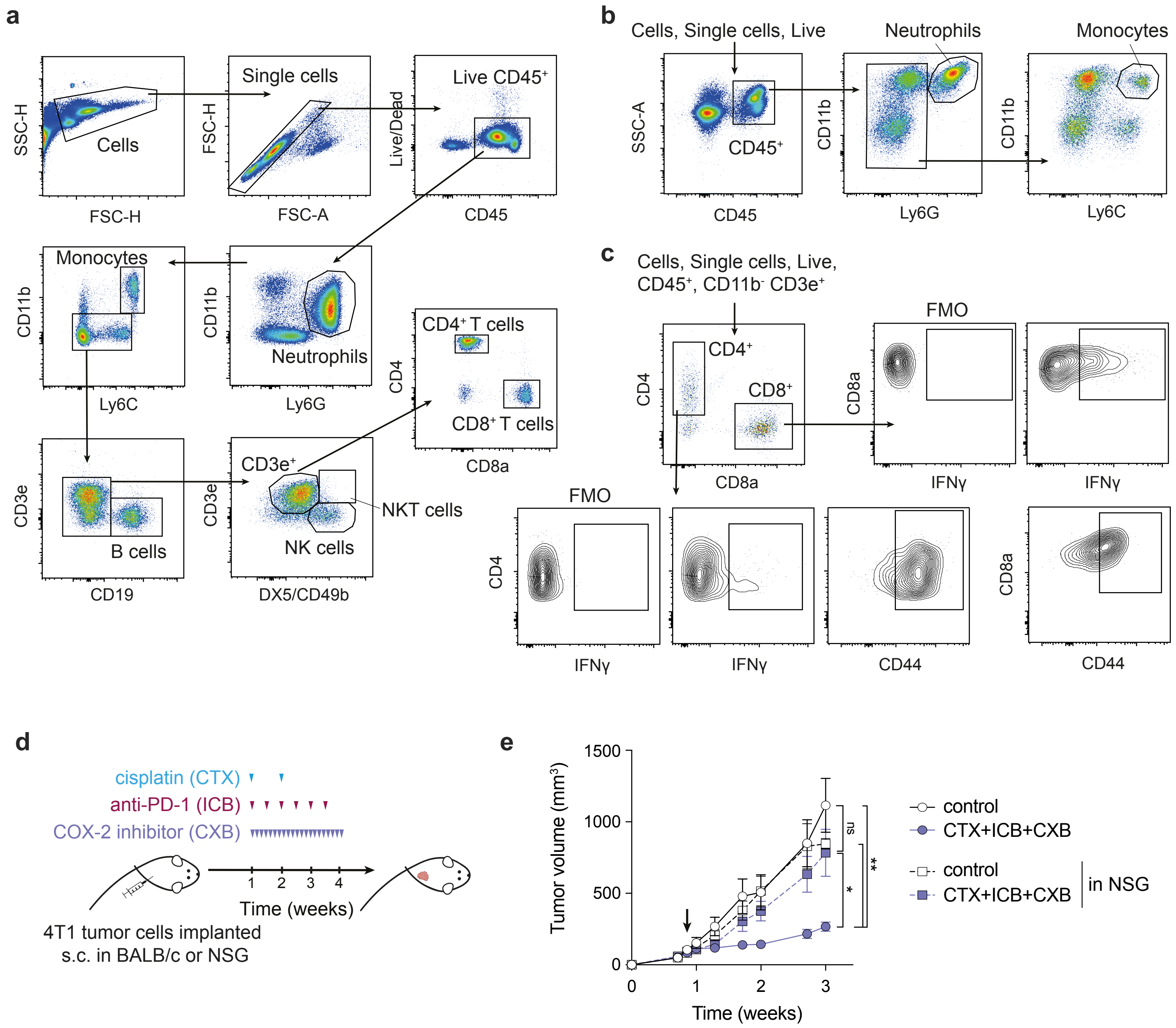

Supplementary Figure 10 (related to Figure 6). Analysis of the immune-dependent effects of the triple combination therapy. Representative flow cytometry gating strategies for immune cell populations in (**a**) peripheral blood two weeks on-treatment and (**b-c**) tumor infiltrating cells three weeks on-treatment in 4T1 tumor-bearing mice shown in Figure 6. (**d**) Immunocompetent BALB/c or immunodeficient NSG mice were inoculated subcutaneously with 4T1 tumor cells and treatment began on day six post-injection when tumor volumes were 92.0 ±7.3mm^3 (mean ±SEM). (**e**) Mean tumor growth profiles of control or triple combination treated mice (mean ±SEM*,* *n* = 5 mice per group). *$p < 0.05$, **$p < 0.01$ as determined by two-way ANOVA. Source data and exact *p* values are provided as a Source Data file.

Supplementary Figure 11

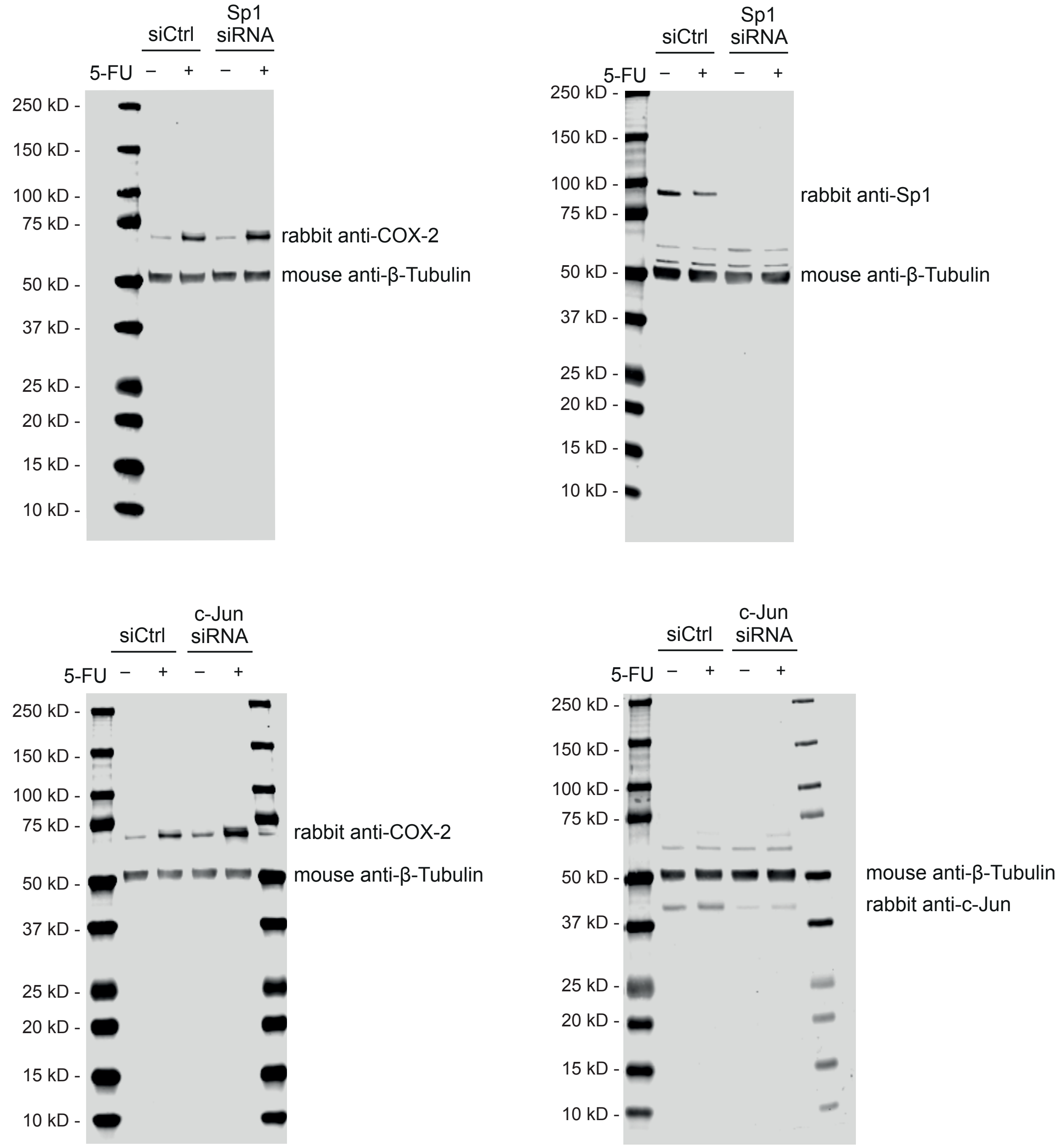

Supplementary Figure 11. Uncropped western blots. Full scans for western blots shown in Supplementary Figure 3e.

Supplementary Table 1

Antineoplastic compound	GFP score	Confluency score
5-fluorouracil	6.25	0.70
Altretamine	1.34	0.91
Amethopterin (R,S)	18.79	0.18
Anastrozole	0.04	0.98
Azacytidine-5	20.97	0.42
Azaguanine-8	3.36	0.73
Azathioprine	1.50	0.95
Beta-Escin	0.15	0.80
Bicalutamide	0.01	1.27
Busulfan	0.35	1.01
Camptothecine (S,+)	8.45	0.38
Carmofur	15.19	0.38
Catharanthine	0.18	0.98
Chlormadinone acetate	0.00	0.90
Chlorotrianisene	0.00	1.21
Cladribine	7.42	0.38
Cyclophosphamide	0.46	0.68
Cyproterone acetate	0.00	1.25
Cytarabine	0.54	0.72
Dacarbazine	2.43	1.15
Daunorubicin hydrochloride	38.66	0.37
Docetaxel	6.21	0.48
Doxorubicin hydrochloride	28.21	0.51
Epirubicin hydrochloride	8.41	0.28
Erlotinib	4.11	0.87
Estramustine	10.33	0.63
Etanidazole	0.00	1.06
Etoposide	0.00	0.91
Exemestane	0.00	0.75
Finasteride	0.00	1.13
Floxuridine	8.46	0.60
Fludarabine	0.00	1.12
Flutamide	0.00	1.16
Formestane	0.15	1.04
Fulvestrant	1.47	0.75
Gefitinib	11.30	0.55
Gemcitabine	11.54	0.47
Glutethimide, para-amino	1.44	0.87
Hesperidin	0.04	1.17
Hexestrol	1.26	0.97
Histamine dihydrochloride	0.57	1.31
Idebenone	0.00	0.50
Ifosfamide	0.00	0.63
Imatinib	0.00	0.94
Iobenguane sulfate	0.00	1.13
Irinotecan hydrochloride trihydrate	0.57	0.57
Letrozole	0.00	1.10
Megestrol acetate	0.02	1.25
Methotrexate	12.05	0.24
Mitotane	0.61	1.10
Mitoxantrone dihydrochloride	20.60	0.34
N6-methyladenosine	0.00	0.61
Nelfinavir mesylate	0.00	0.92
Nilutamide	0.00	0.81
Nocodazole	35.76	0.30
Paclitaxel	10.49	0.32
Pemetrexed disodium	8.25	0.72
Procarbazine hydrochloride	0.66	1.04
Raltitrexed	8.11	0.72
Streptozotocin	1.15	1.03
Tamoxifen citrate	6.16	0.73
Tegafur	0.44	1.11
Temozolomide	1.29	1.10
Thioguanosine	0.00	1.04
Topotecan	17.87	0.35
Toremifene	1.82	0.79
Ubenimex	0.60	0.98
Vatalanib	0.22	1.05
Vorinostat	32.63	0.44

Supplementary Table 1 (related to Figure 3). List of antineoplastic agents in the compound library screen with corresponding GFP and confluency scores.
