# Supplementary material for: Chemotherapy-induced COX-2 upregulation by cancer cells defines their inflammatory properties and limits the efficacy of chemoimmunotherapy combinations
Source: Nat Commun. 2022 Apr 19;13:2063. doi: 10.1038/s41467-022-29606-9 (PMC9018752; doi:10.1038/s41467-022-29606-9)
Supplement: Supplementary file 2 — Reporting Summary [file 41467_2022_29606_MOESM2_ESM.pdf]

nature portfolio

Corresponding author(s): Santiago Zelenay

Last updated by author(s): Mar 14, 2022

Reporting Summary

Nature Portfolio wishes to improve the reproducibility of the work that we publish. This form provides structure for consistency and transparency in reporting. For further information on Nature Portfolio policies, see our Editorial Policies and the Editorial Policy Checklist.

Statistics

For all statistical analyses, confirm that the following items are present in the figure legend, table legend, main text, or Methods section.

n/a	Confirmed	
☐	☒	The exact sample size (n) for each experimental group/condition, given as a discrete number and unit of measurement
☐	☒	A statement on whether measurements were taken from distinct samples or whether the same sample was measured repeatedly
☐	☒	The statistical test(s) used AND whether they are one- or two-sided<br>*Only common tests should be described solely by name; describe more complex techniques in the Methods section.*
☒	☐	A description of all covariates tested
☐	☒	A description of any assumptions or corrections, such as tests of normality and adjustment for multiple comparisons
☐	☒	A full description of the statistical parameters including central tendency (e.g. means) or other basic estimates (e.g. regression coefficient) AND variation (e.g. standard deviation) or associated estimates of uncertainty (e.g. confidence intervals)
☐	☒	For null hypothesis testing, the test statistic (e.g. F, t, r) with confidence intervals, effect sizes, degrees of freedom and P value noted<br>*Give P values as exact values whenever suitable.*
☒	☐	For Bayesian analysis, information on the choice of priors and Markov chain Monte Carlo settings
☒	☐	For hierarchical and complex designs, identification of the appropriate level for tests and full reporting of outcomes
☐	☒	Estimates of effect sizes (e.g. Cohen's d, Pearson's r), indicating how they were calculated

Our web collection on statistics for biologists contains articles on many of the points above.

Software and code

Policy information about availability of computer code

Data collection	ELISA data was acquired on Spectra Max M5 plate reader (Molecular Devices)<br>Western blot bands were visualized using Odyssey CLx system (Li-COR)<br>qPCR data was acquired on QuantStudio 5 system (Applied Biosystems)<br>Flow cytometry samples were acquired on a NovoCyte (ACEA) or LSRFortessa X-20 (BD Biosciences)<br>Live-cell imaging was performed using an Incucyte S3 (Essen BioScience)<br>Multiplex cytokine analysis was performed using MAGPIX (Luminex)<br><br>Graphics in Figure 1g, 3a and 3e were made using Biorender
Data analysis	Data were analyzed using Microsoft Excel 2019 and/or GraphPad Prism (v9.1.2), Incucyte software (GUI v2020C Rev1), FlowJo (v10.8.0) and Image Studio Lite (v5.2.5)

For manuscripts utilizing custom algorithms or software that are central to the research but not yet described in published literature, software must be made available to editors and reviewers. We strongly encourage code deposition in a community repository (e.g. GitHub). See the Nature Portfolio guidelines for submitting code & software for further information.

Data

Policy information about availability of data

All manuscripts must include a data availability statement. This statement should provide the following information, where applicable:

- Accession codes, unique identifiers, or web links for publicly available datasets
- A description of any restrictions on data availability
- For clinical datasets or third party data, please ensure that the statement adheres to our policy

The NCI-60 human cancer cell publically available data used in this study are available in the NCBI Gene Expression Omnibus database under accession code GSE116436. Source data are provided with this paper. The relevant data supporting the findings in this study are available in the Article, Supplementary Information or Source Data file.

Field-specific reporting

Please select the one below that is the best fit for your research. If you are not sure, read the appropriate sections before making your selection.

[x] Life sciences [ ] Behavioural & social sciences [ ] Ecological, evolutionary & environmental sciences

For a reference copy of the document with all sections, see nature.com/documents/nr-reporting-summary-flat.pdf

Life sciences study design

All studies must disclose on these points even when the disclosure is negative.

Sample size	No statistical analysis was performed to predetermine sample size. The sample size was chosen based on previous published studies including our own (Bonavita et al, 2020; Pelly et al, 2021).<br>All sample size, statistical tests and p-values are indicated in the figure legends and described in the methods.
Data exclusions	No data were excluded from analysis
Replication	All experiments were repeated at least twice with similar results. Figure legends specify where data shown is representative of technical replicates, independent experiments or for in vivo experiments individual mice pooled across independent experiments.
Randomization	Stratified randomization was applied in order to normalize tumor sizes and body weights across treatment groups for in vivo experiments. All mice were assigned randomly into groups for peritoneal lavage experiments.
Blinding	The investigators were not blinded to allocation of animals during experiments and outcome assessments. The counting of macroscopic lung metastases was performed by an investigator blinded to treatment group. The remaining experiments were not blinded as the investigators who set up the experiment analyzed the data, which is incompatible with blinding.

Reporting for specific materials, systems and methods

We require information from authors about some types of materials, experimental systems and methods used in many studies. Here, indicate whether each material, system or method listed is relevant to your study. If you are not sure if a list item applies to your research, read the appropriate section before selecting a response.

Materials & experimental systems

n/a	Involved in the study	
[ ]	[x]	Antibodies
[ ]	[x]	Eukaryotic cell lines
[x]	[ ]	Palaeontology and archaeology
[ ]	[x]	Animals and other organisms
[x]	[ ]	Human research participants
[x]	[ ]	Clinical data
[x]	[ ]	Dual use research of concern

Methods

n/a	Involved in the study	
[x]	[ ]	ChIP-seq
[ ]	[x]	Flow cytometry
[x]	[ ]	MRI-based neuroimaging

Antibodies

Antibodies used	in vivo dosing: anti-PD-1 (clone RMP1-14, #BE0146, lot #717919M1 BioXCell) diluted to 2 mg/ml and administered at 200 µg per 100 µl i.p. injection<br><br>Western blot primary antibodies (Cell Signaling Technology):<br>COX-2 1:1000 (clone D5H5, #12282)

β-Tubulin 1:2000 (clone D3U1W, #86298)
NF-κB p65 1:2000 (clone D14E12, #8242)
c-Jun 1:1000 (clone 60A8, #9165)
β-Actin 1:4000 (clone D6A8, #8457)

Western blot primary antibodies:
c/EBPβ 1:500 (H-7, Santa Cruz #sc-7962)
Sp1 1:10000 (Novus Biologicals, #600-232)

Western blot secondary antibodies (Li-COR):
IRDye 680RD Goat Anti-Mouse IgG 1:15000 (#926-68070)
IRDye 800CW Goat Anti-Rabbit IgG 1:15000 (#926-32211)

Flow cytometry antibodies:
anti-CD16/32 (1:250, clone 93, eBioscience, #16-0161-85)
CD45-BV605 (1:200, clone 30-F11, BioLegend #103140)
CD11b-BV785 (1:300, clone M1/70, BioLegend, #101243)
Ly6G-PE/Dazzle 594 (1:400, clone 1A8, BioLegend, #127648)
Ly6G-FITC (1:400, clone 1A8, BioLegend, #127606)
Ly6C-FITC (1:400, clone AL-21, BD Biosciences, #553104)
Ly6C-BV421 (1:400, clone HK1.4, BioLegend, #128032)
F4/80-PE-Cy7 (1:100 or 1:200, clone BM8, BioLegend, #123114)
CD11c-AlexaFluor700 (1:200, clone N418, BioLegend, #117320)
MHCII I-A/I-E APC-eFluor780 (1:300, clone M5/114.15.2, eBioscience, #47-5321-82)
MHCII I-A/I-E-PerCP-Cy5.5 (1:300, clone M5/114.15.2, BioLegend, #107626)
CD274(PD-L1)-PE (1:100, clone MIH5, eBioscience, #12-5982-82)
Siglec-F-BV711 (1:100, clone E50-2440, BD Biosciences, #740764)
CD19-PE (1:400, clone 1D3, BD Biosciences, #557399)
CD49b-APC (1:100, clone DX5, BioLegend, #108910)
CD3ε-PerCP-Cy5.5 (1:100, clone 145-2C11, eBioscience, #45-0031-82)
CD3ε-PE/Dazzle 594 (1:200, clone 145-2C11, BioLegend, #100348)
CD8α-PE (1:100, clone 53-6.7, eBioscience, #12-0081-82)
CD8α-PE-Cy7 (1:200, clone 53-6.7, BioLegend, #100722)
CD4-FITC (1:300, clone RM4-5, BioLegend, #100510)
CD44-APC-eFluor780 (1:100, clone IM7, eBioscience, #47-0441-82)
IFNγ-eFluor450 (1:80, clone XMG1.2, eBioscience, #48-7311-82)

Validation

All antibodies are from commercial sources and have been validated by the vendors, with validation data available on the manufacturer's website:

anti-PD-1 (https://bxcell.com/product/invivomab-anti-m-pd-1/)
anti-COX-2 (https://www.cellsignal.co.uk/products/primary-antibodies/cox2-d5h5-xp-rabbit-mab/12282)
anti-β-Tubulin (https://www.cellsignal.co.uk/products/primary-antibodies/b-tubulin-d3u1w-mouse-mab/86298)
anti-NF-κB p65 (https://www.cellsignal.co.uk/products/primary-antibodies/nf-kb-p65-d14e12-xp-rabbit-mab/8242)
anti-c-Jun (https://www.cellsignal.co.uk/products/primary-antibodies/c-jun-60a8-rabbit-mab/9165)
anti-β-Actin (https://www.cellsignal.co.uk/products/primary-antibodies/b-actin-d6a8-rabbit-mab/8457)
anti-c/EBPβ (https://www.scbt.com/p/c-ebp-beta-antibody-h-7?requestFrom=search)
anti-Sp1 (https://www.novusbio.com/products/sp1-antibody_nb600-232)
IRDye 680RD Goat Anti-Mouse IgG (https://www.licor.com/bio/reagents/irdye-680rd-goat-anti-mouse-igg-secondary-antibody)
IRDye 800CW Goat Anti-Rabbit IgG (https://www.licor.com/bio/reagents/irdye-800cw-goat-anti-rabbit-igg-secondary-antibody)
anti-CD16/32 (https://www.thermofisher.com/antibody/product/CD16-CD32-Antibody-clone-93-Monoclonal/16-0161-82)
CD45-BV605 (https://www.biolegend.com/en-gb/products/brilliant-violet-605-anti-mouse-cd45-antibody-8721)
CD11b-BV785 (https://www.biolegend.com/en-us/products/brilliant-violet-785-anti-mouse-human-cd11b-antibody-7958)
Ly6G-PE/Dazzle 594 (https://www.biolegend.com/en-us/search-results/pe-dazzle-594-anti-mouse-ly-6g-antibody-12246)
Ly6G-FITC (https://www.biolegend.com/en-us/products/fitc-anti-mouse-ly-6g-antibody-4775)
Ly6C-FITC (https://www.bdbiosciences.com/en-us/products/reagents/flow-cytometry-reagents/research-reagents/single-color-antibodies-ruo/fitc-rat-anti-mouse-ly-6c.561085)
Ly6C-BV421 (https://www.biolegend.com/nl-nl/products/brilliant-violet-421-anti-mouse-ly-6c-antibody-8586)
F4/80-PE-Cy7 (https://www.biolegend.com/en-us/products/pe-cyanine7-anti-mouse-f4-80-antibody-4070?GroupID=BLG5319)
CD11c-AlexaFluor700 (https://www.biolegend.com/en-gb/products/alexa-fluor-700-anti-mouse-cd11c-antibody-3429)
MHCII I-A/I-E APC-eFluor780 (https://www.thermofisher.com/antibody/product/MHC-Class-II-I-A-I-E-Antibody-clone-M5-114-15-2-Monoclonal/47-5321-82)
MHCII I-A/I-E-PerCP-Cy5.5 (https://www.biolegend.com/en-us/search-results/percp-cyanine5-5-anti-mouse-i-a-i-e-antibody-4282)
CD274(PD-L1)-PE (https://www.thermofisher.com/antibody/product/CD274-PD-L1-B7-H1-Antibody-clone-MIH5-Monoclonal/12-5982-82)
Siglec-F-BV711 (https://www.bdbiosciences.com/en-us/products/reagents/flow-cytometry-reagents/research-reagents/single-color-antibodies-ruo/bv711-rat-anti-mouse-siglec-f.740764)
CD19-PE (https://www.bdbiosciences.com/en-eu/products/reagents/flow-cytometry-reagents/research-reagents/single-color-antibodies-ruo/pe-rat-anti-mouse-cd19.557399)

CD49b-APC (https://www.biolegend.com/en-us/products/apc-anti-mouse-cd49b-pan-nk-cells-antibody-231?GroupID=BLG4768)
CD3ε-PerCP-Cy5.5 (https://www.thermofisher.com/antibody/product/CD3e-Antibody-clone-145-2C11-Monoclonal/45-0031-82)
CD3ε-PE/Dazzle 594 (https://www.biolegend.com/en-gb/products/pe-dazzle-594-anti-mouse-cd3epsilon-antibody-10066
CD8α-PE (https://www.thermofisher.com/antibody/product/CD8a-Antibody-clone-53-6-7-Monoclonal/12-0081-82)
CD8α-PE-Cy7 (https://www.biolegend.com/en-us/products/pe-cyanine7-anti-mouse-cd8a-antibody-1906?GroupID=BLG2559)
CD4-FITC (https://www.biolegend.com/en-us/products/fitc-anti-mouse-cd4-antibody-480?GroupID=BLG4745)
CD44-APC-eFluor780 (https://www.thermofisher.com/antibody/product/CD44-Antibody-clone-IM7-Monoclonal/47-0441-82)
IFNγ-eFluor450 (https://www.thermofisher.com/antibody/product/IFN-gamma-Antibody-clone-XMG1-2-Monoclonal/48-7311-82)

Eukaryotic cell lines

Policy information about cell lines

Cell line source(s)	Murine cancer cell lines: 4T1 breast, CT26 colorectal, MC38 colorectal, E0771 breast and 3LL lung are commercially available; 5555 and 4434 melanoma were obtained from Dr Richard Marais; TB32908 pancreatic, TB32043 pancreatic, TB32047 pancreatic were obtained from Dr Kris Freese and YUMM1.1 were obtained from Dr Marcus Bosenberg. The human cancer cell lines: NCI-H2122 lung carcinoma and MCF7 breast carcinoma cells are commercially available.
Authentication	None of the cell lines used were authenticated
Mycoplasma contamination	All cell lines were routinely tested and confirmed to be negative for mycoplasma
Commonly misidentified lines (See ICLAC register)	No commonly misidentified cell lines were used

Animals and other organisms

Policy information about studies involving animals; ARRIVE guidelines recommended for reporting animal research

Laboratory animals	All mice were maintained under pathogen-free conditions in ventilated cages with environment enrichment in the Biological Resources Unit at the CRUK Manchester Institute (CRUK MI), and allowed free access to irradiated food and autoclaved water ad libitum in a 12h light/dark cycle, with room temperature at 21 ± 2 °C and a humidity of 45-65%. 6-12 week old female BALB/c mice (Envigo) and 12 week old female NSG mice (Charles River) were used in experiments. Mice were age-matched in experiments.
Wild animals	No wild animals were used
Field-collected samples	There are no field-collected samples
Ethics oversight	All procedures involving animals were performed in accordance with the PDCC31AAF license approved by the Animal Welfare and Ethical Review Bodies (AWERB) of the CRUK Manchester Institute, and in accordance with National Home Office regulations under the Animals (Scientific Procedures) Act 1986

Note that full information on the approval of the study protocol must also be provided in the manuscript.

Flow Cytometry

Plots

Confirm that:

- [x] The axis labels state the marker and fluorochrome used (e.g. CD4-FITC).
- [x] The axis scales are clearly visible. Include numbers along axes only for bottom left plot of group (a 'group' is an analysis of identical markers).
- [x] All plots are contour plots with outliers or pseudocolor plots.
- [x] A numerical value for number of cells or percentage (with statistics) is provided.

Methodology

Sample preparation	For analysis of peripheral blood leukocytes, 50µl peripheral blood was taken from mice via tail-vein into an EDTA-coated capillary and then 1.5ml tubes on ice. Samples were centrifuged at 300 rcf for 6 min at 4˚C to separate plasma and cells, FACS buffer was added to the cell pellet and cell suspensions were moved to a 96-well V-bottom plate for antibody staining. Red blood cells were lysed using ACK buffer for 1min (Gibco).<br><br>For analysis of tumor-infiltrating leukocytes, tumors were collected into complete RPMI on ice. The surface of tumor samples were dried with paper and weights recorded. Samples were transferred into C-tubes (Miltenyi Biotech) containing RPMI and Collagenase IV (200 U/ml, Worthington Biochemical) and DNase I (0.2 mg/ml, Roche), then minced using scissors. The C-tubes were placed in a GentleMACS Octo Dissociator (Miltenyi Biotech), and tumors disaggregated with 2 rounds of the automated program m_impTumor_02_01. Dissociated tumors were incubated for 30 min at 37°C and disaggregated for one more round. The C-tubes were centrifuged and pellets resuspended in cold complete RPMI before being filtered through a 70µM cell strainer and pelleted. Cell suspensions were resuspended in FACS buffer. Fc receptors were saturated with anti-CD16/32 (clone 93, eBioscience) 5 min before staining. Cell viability was determined by Aqua LIVE/Dead-405nm staining

(Invitrogen). For intracellular cytokine detection, cells were stimulated ex vivo for 4h with Cell Stimulation Cocktail (ThermoFisher) and stained using the Intracellular Fixation & Permeabilization Buffer Set (eBioscience) following manufacturer instructions. Monensin and Brefeldin A (both eBioscience) were added 2h before the staining and non-specific binding of intracellular epitopes was blocked by pre-incubation of cells with 2% Normal Rat Serum (ThermoFisher).

For analysis of peritoneal lavage, cells were pelleted by centrifugation and resuspended in FACS buffer (PBS containing 1% FBS and 0.01% sodium azide) before filtering through a 70µm cell strainer. Fc receptors were saturated with anti-CD16/32 (clone 93, eBioscience) 5 min before staining. Cell viability was determined by Aqua LIVE/Dead-405nm staining (Invitrogen).

Instrument	BD LSRFortessa X-20 and ACEA NovoCyte
Software	FlowJo v10.8.0
Cell population abundance	Cells were not sorted for downstream analysis
Gating strategy	Gating strategies are provided in the Supplementary Information. Briefly, debris was excluded based on FSC-A and SSC-A. Single cells were gated based on FSC-H and FSC-A. Live cells were gated based on cells which were negative for the Aqua LIVE/Dead-405nm (Invitrogen) stain. Immune cells were then gated on by CD45+ staining and sub-populations defined as per the gating strategies.

☒ Tick this box to confirm that a figure exemplifying the gating strategy is provided in the Supplementary Information.
